# Supplementary material for: Cryopreserved Human Oocytes and Cord Blood Cells Can Produce Somatic Cell Nuclear Transfer-Derived Pluripotent Stem Cells with a Homozygous HLA Type
Source: Stem Cell Reports. 2020 Jun 4;15(1):171–84. doi: 10.1016/j.stemcr.2020.05.005 (PMC7363744; doi:10.1016/j.stemcr.2020.05.005)
Supplement: Document S1. Supplemental Experimental Procedures and Figures S1–S7 [file mmc1.pdf]

Stem Cell Reports, Volume *15*

Supplemental Information

Cryopreserved Human Oocytes and Cord Blood Cells Can Produce Somatic Cell Nuclear Transfer-Derived Pluripotent Stem Cells with a Homozygous HLA Type

Jeoung Eun Lee, Ji Yoon Lee, Chang-Hwan Park, Jin Hee Eum, Soo Kyung Jung, A-Reum Han, Dong-Won Seol, Jin Saem Lee, Hyun Soo Shin, Jung Ho Im, Taehoon Chun, Kyungsoo Ha, Deok Rim Heo, Tae Ki Yoon, and Dong Ryul Lee

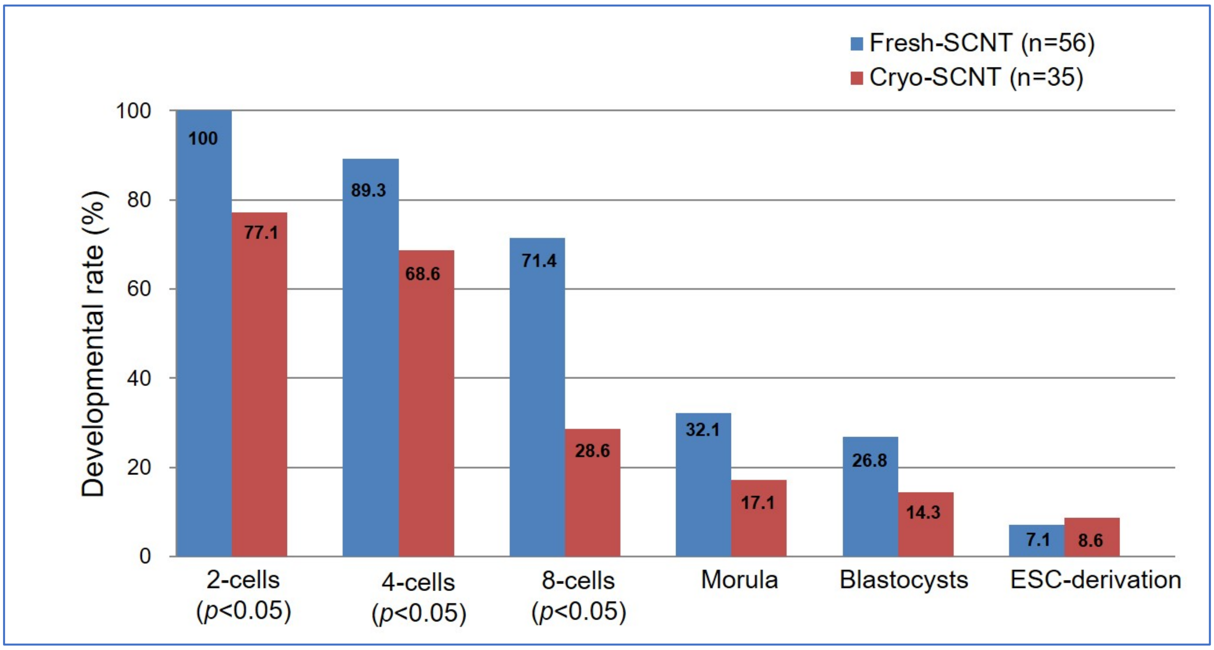

Figure S1. Comparison of embryonic development between fresh oocyte-based (Fresh)-somatic cell nuclear transfer (SCNT) and cryopreserved oocyte-based (Cryo)-SCNT (related to Figure 1)

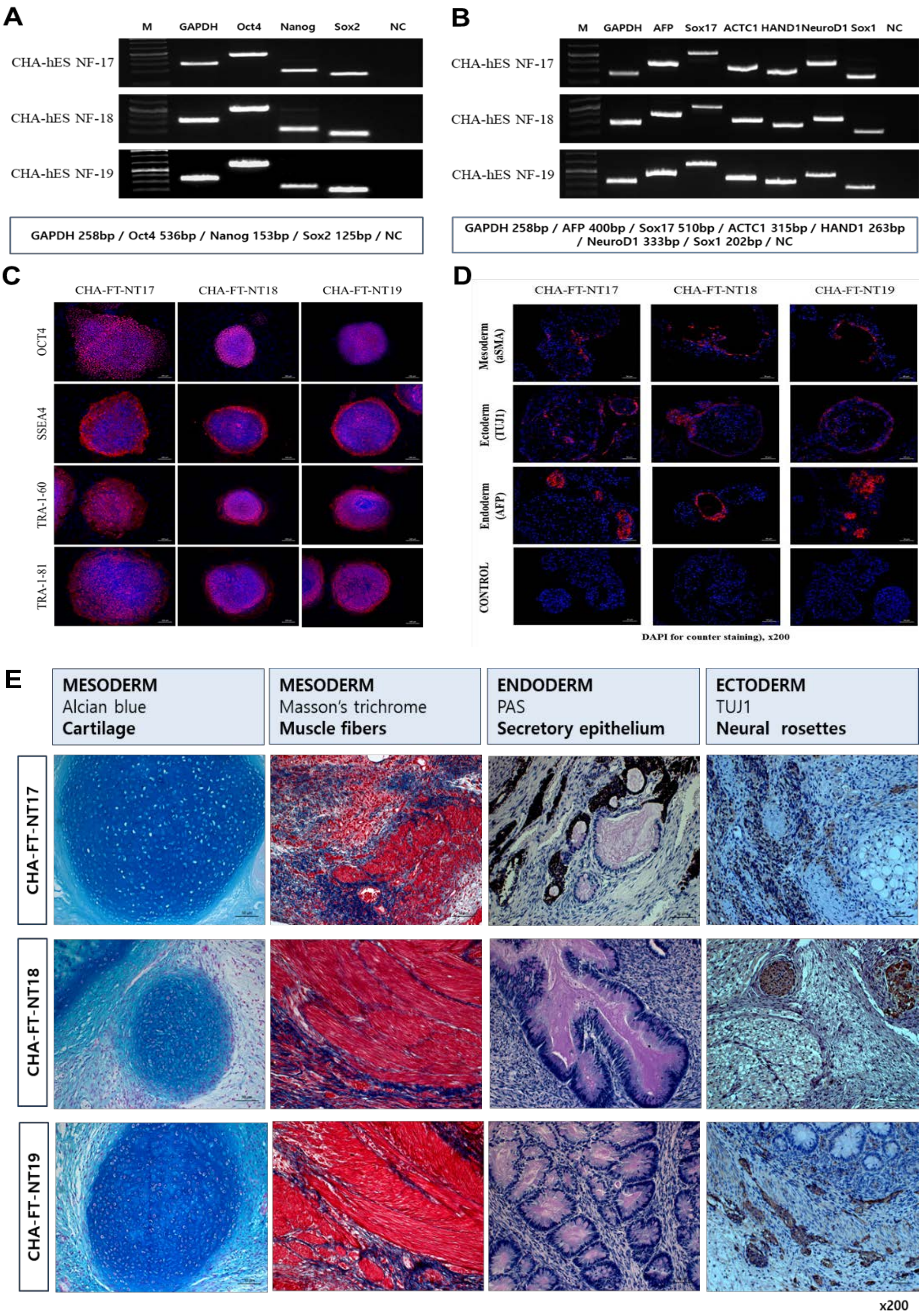

Figure S2. Characteristics of somatic cell nuclear transfer-derived pluripotent stem cells (SCNT-PSCs) obtained using cryopreserved human oocytes (related to Figure 2) To confirm the pluripotency of the

SCNT-PSC line derived from vitrified/warmed oocytes, their differentiation capacity was examined by *in vitro* EB formation. (A) RT-PCR showing the expression of pluripotency-related genes (*POU5F1*(*OCT4)*, *SOX2*, and *NANOG*) in SCNT-PSC colonies. (B) RT-PCR showing the expression of three germ layer-marker genes in embryonic bodies (EBs) of SCNT-PSCs. *GAPDH*: internal control; *SOX17* and *HAND1:* mesoderm ectoderm; *NEUROD1* and *SOX1*: ectoderm; *AFP* and *ACTC1*: endoderm; and N.C: negative control. (C) Expression of pluripotent stem cell markers (OCT4, SSEA4, TRA-1-60, and TRA-1-81) in SCNT-PSC colonies. The scale bars indicate 100µm. (D) Expression of markers of three germ layers in EBs of SCNT-PSCs, αSMA: mesoderm; TUJ1: ectoderm; AFP: endoderm; and control (no antibody). The scale bars indicate 50µm. (E) To confirm the pluripotency of the SCNT-PSC line derived from vitrified/warmed oocytes, their differentiation capacity was examined by *in vivo* teratoma assays. Teratoma confirmed the differentiation of somatic cell nuclear transfer-derived pluripotent stem cells (SCNT-PSCs) using cryopreserved human oocytes. From left, Alcian blue staining of mesoderm-derived cartilage, Masson’s trichrome staining of mesoderm-derived muscle fibers, periodic acid Schiff (PAS) staining of endoderm-derived secretory epithelium, and TUJ1 staining of ectoderm-derived neural rosettes. The scale bars indicate 50µm.

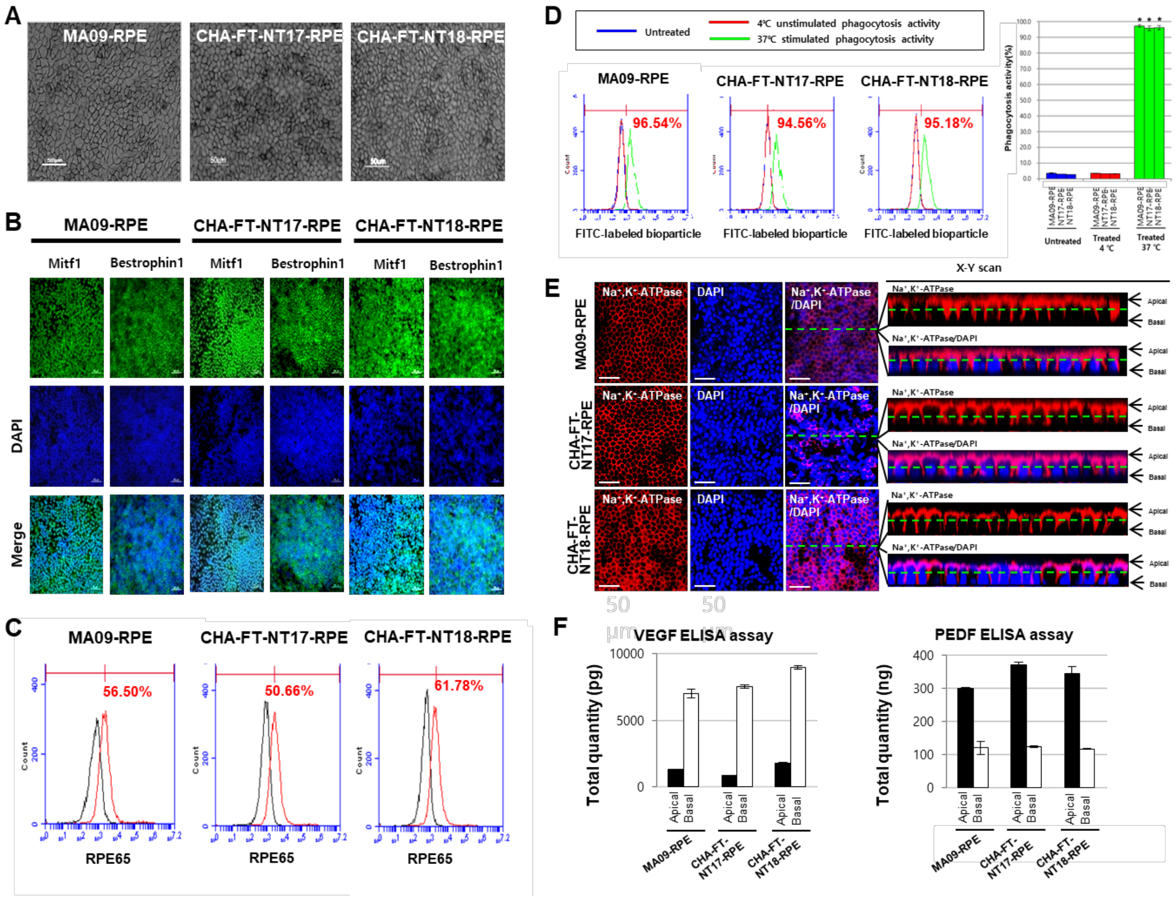

Figure S3. Characterization of somatic cell nuclear transfer-pluripotent stem cell-derived retinal pigmented epithelial cells (SCNT-PSC-RPEs) (related to Figure 3) (A) SCNT-PSC-RPEs (CHA-FT-NT17-RPEs and CHA-FT-NT18-RPEs) and a well-characterized hESC line (MA09-RPEs) have shown typical RPE characteristics, such as pigmentation and cuboidal epithelial morphology in tissue culture. The scale bars indicate 50μm. (B, C) Compared to MA09-RPEs, SCNT-PSC-RPEs were similarly stained for hRPE markers, including MITF1 and BESTROPHIN1, and they expressed RPE65. The scale bars indicate 50μm. (D-F) The phagocytotic activity and polarity (Na^+, K^+-ATPase activity and expression of growth factors) of RPEs differentiated from PSCs were not different. The scale bars indicate 50μm.

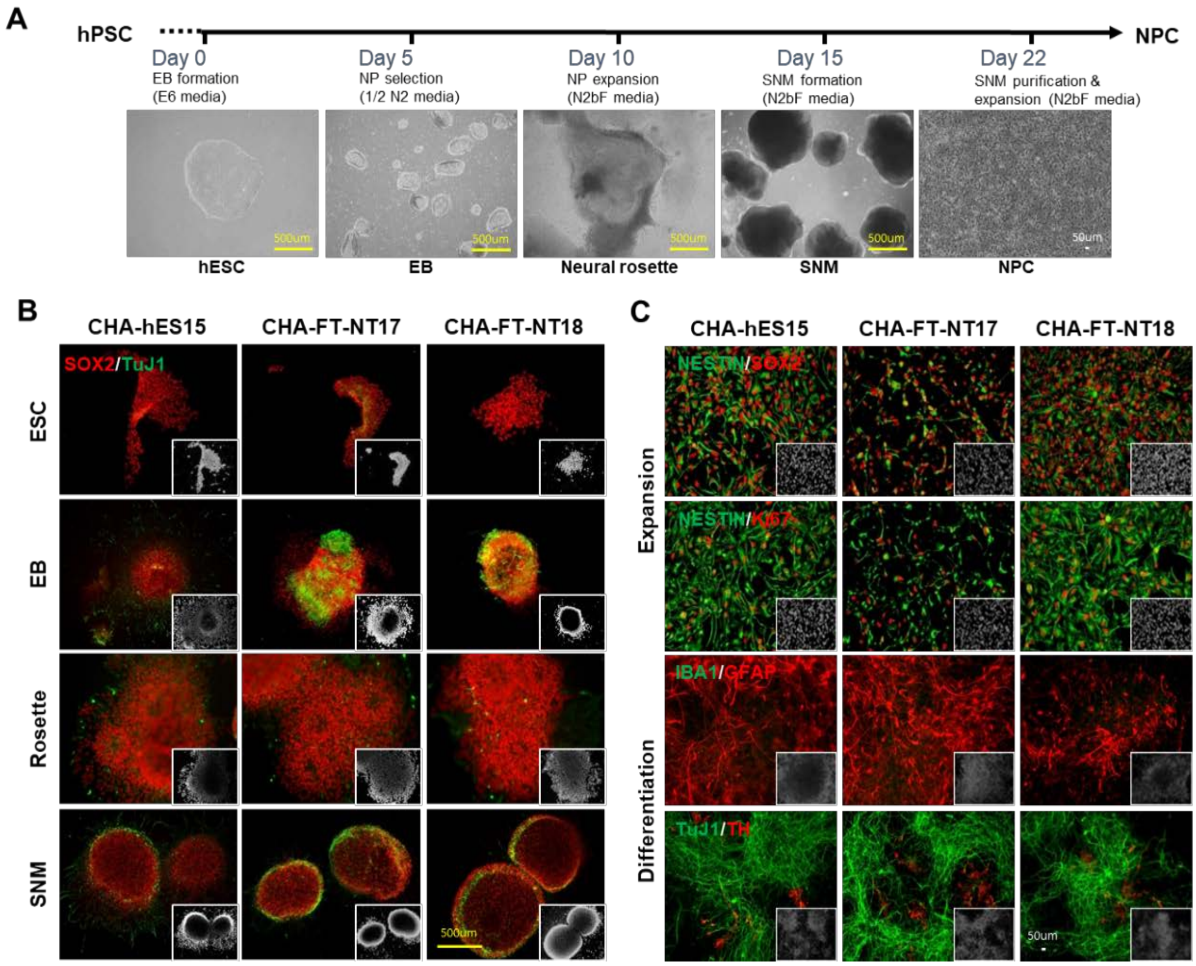

Figure S4. Characterization of somatic cell nuclear transfer-pluripotent stem cell-derived neural precursor cells (SCNT-PSC-NPCs) (related to Figure 3) (A) Schematic procedures for the *in vitro* differentiation of human PSCs into NPCs via spherical neural masses (SNMs). (B) Highly homogeneous SNMs were generated regardless of the human PSC line. Although some of the embryonic bodies expressed TuJ1, a neuronal marker, this expression disappeared in rosette-like structures and SNMs, indicating that rosettes and SNMs have pure NPC properties. (C) Differentiation characteristics of NPCs derived from various human PSCs. Most of the proliferating NPCs expressed NESTIN, SOX2 and Ki67. Upon removal of bFGF, NPCs differentiated into astrocytes and neurons as well as dopamine neurons. The yellow scale bars indicate 500µm. The white scale bars indicate 50µm.

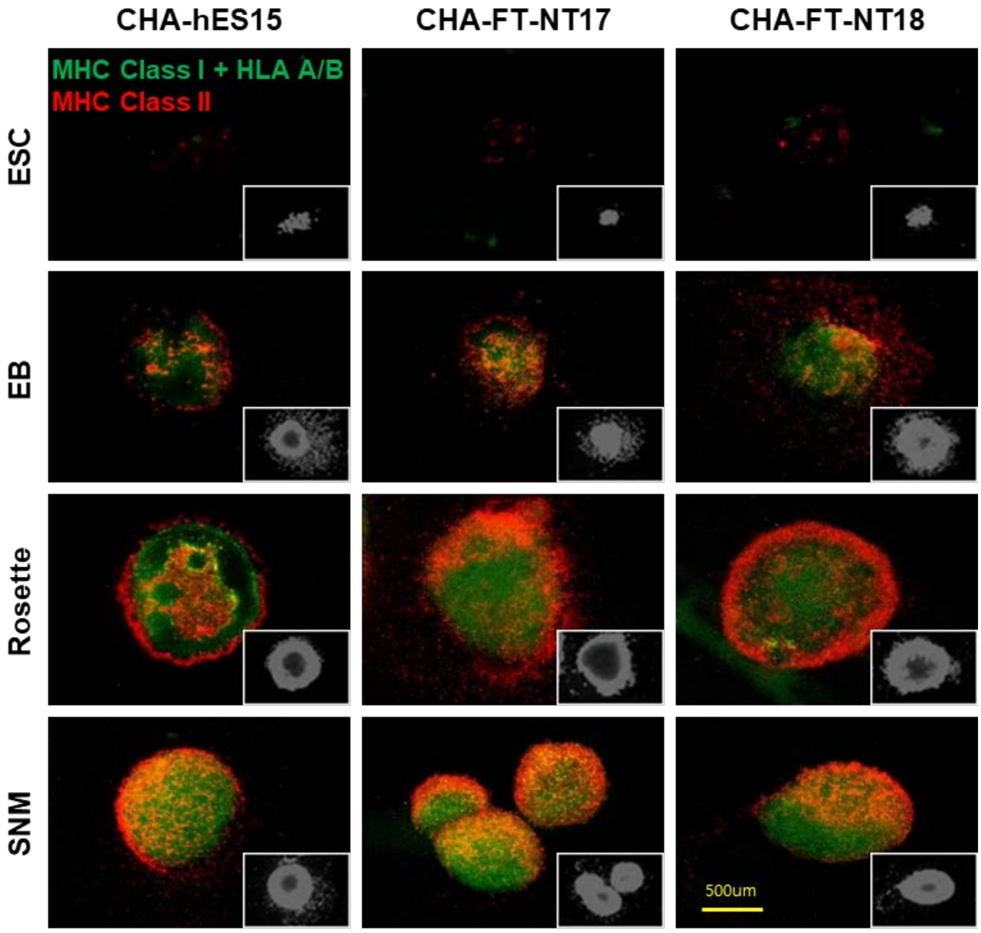

Figure S5. Expression of major histocompatibility antigens by differentiation stage in human pluripotent stem cells (PSCs) (related to Figure 4) MHC class I and II antigens were not expressed in PSCs regardless of the cell line, but their expression gradually increased with differentiation into the embryonic body (EB), neural rosette, and spherical neural mass (SNM) stages. The scale bar indicates 500μm.

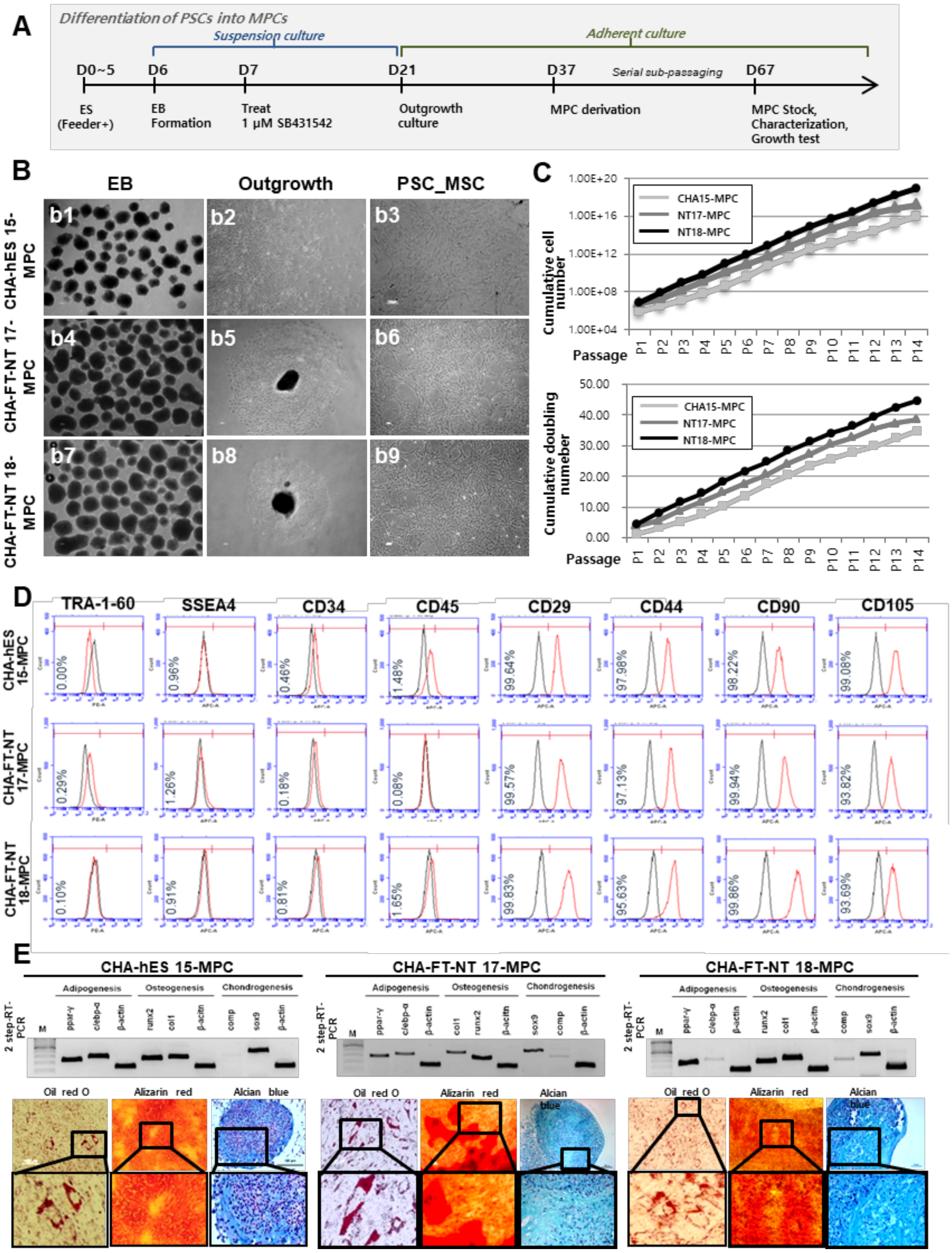

Figure S6. Generation and characterization of mesenchymal progenitor cells (MPCs) from human somatic cell nuclear transfer-pluripotent stem cells (SCNT-PSCs) (related to Figure 6) (A) Schema outlining the PSC-MPC differentiation protocol. The black horizontal line: time-line of days after initiation of differentiation. Days when a treatment was introduced are indicated (see Methods for details). From day 0 (D0) to day 6 (D6), human PSCs were cultured with MEF feeder cells for EB formation. SB431542 (1 μM) was added from D7 until D21 and then, these EBs were attached to the 6-well culture plate for outgrowth and expansion of MPCs until D37. Through serial subpassaging, matured and purified PSC-MPC were obtained and collected for the proliferation test, FACS analysis, and differentiation into specific-mesodermal lineages (adipocytes, osteocytes, and chondrocytes). (B) Representative morphology of human SCNT-PSC-derived MPCs (SCNT-PSC-MPCs) at different stages during the course of differentiation. (b1,b4,b7) Embryoid bodies (EBs) on day 1. (b2,b5,b8) Attachment and outgrowth of primitive MPCs on

day 14. (b3,b6,b9) The typical morphology of human SCNT-PSC-MPCs at passage 6. The scale bars indicate 100µm. (C) SCNT-PSC-MPC growth kinetics over long-term expansion. Graphs represent the cumulative cell number (upper) and cumulative population doubling level (CPDL) (bottom). Graphs are displayed from the passage in the initiation state of MPC-derivation to the present passage (P1 to P14). (d) Surface antigen profiling by FACS in SCNT-PSC-MPCs for TRA-1-60 and SSEA4 used as pluripotent markers, CD34 and CD45 used as hematopoietic markers, and CD29, CD44, CD90, and CD105 used as mesenchymal progenitor cell markers. (E) The transcription levels of the adipocyte genes, *PPAR-γ* and *C/EBP-α*, the osteocyte genes, *RUNX2* and *COL1*, and the chondrocyte genes, *sox9* and *comp* were measured by RT-PCR. β-actin was used as a loading control (upper). Differentiation ability of SCNT-PSC-MPCs to adipocytes, chondrocytes, and osteocytes (bottom). The lipid droplets in adipocytes were stained with Oil Red O. The calcium deposits in the intracellular region in osteocytes were stained with alizarin red solution. Mucopolysaccharides and acidic mucins expressed on the surface of the chondrocytes were stained with alcian blue solutions. The magnified images of each differentiation are presented in the bottom row. The scale bars indicate 100µm.

A

		HLA-A	HLA-B	HLA-C	DRB1	DQB1	DPB1
	SCNT-ES derived osteoblast	*33:03(A33)/*33:03(A33)	*44:03(B44)/*44:03(B44)	*14:03/*14:03	*13:.02/*13:02	*06:.04/*06:.04	*04:01/*04:01
Mismatched group for HLA-II DPB1	PBMC 2 (18-15)	*33:03(A33)/*33:03(A33)	*44:03(B44)/*58:01(B58)	*03:02/*14:.03	*13:02/*13:02	*06:04/*06:.09	*02:01/*02:01
	PBMC 3 (16-206)	*02:01(A2)/*33:03(A33)	*27:05(B27)/*44:03(B44)	*01:02/*14:.03	*01:01/*13:02	*05:01/*06:.04	*05:01/*05:01
	Matched group for HLA-I (A,B,C) and II (DRB1, DQB1)						Mismatched for DPB1
Matched group for HLA-II DPB1	PBMC 5 (16-203)	*03:01(A3)/*33:03(A33)	*27:05(B27)/*44:03(B44)	*02:02/*14:.03	*13:02/*15:.02	*06:01/*06:.04	*04:01/*14:01
	PBMC 6 (16-242)	*02:01(A2)/*33:03(A33)	*35:01(B7)/*44:03(B44)	*03:03/*14:.03	*08:03/*13:02	*06:01/*06:.04	*04:01/*19:01
	Matched group for HLA-I (A,B,C) and II (DRB1, DQB1)						Matched for DPB1, thus all ma tched

B

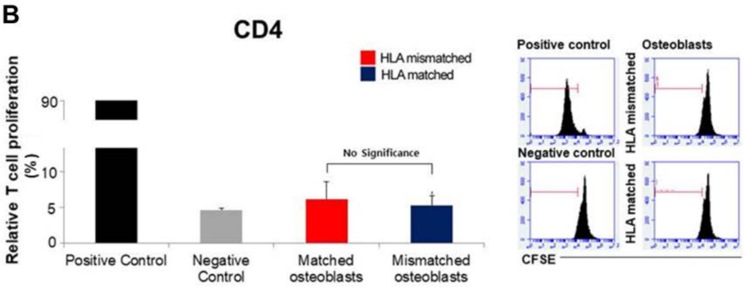

Figure S7. No significant difference was detected in CD4 T cell proliferation, when mismatched HLA-II, DPB1 was cocultured with homozygous SCNT-PSC-derived osteoblasts (related to Figure 6). (A) HLA genotyping data of PBMCs used in the present study. (B) CD4 T cell proliferation data showed that no significant difference by mismatched HLA-II DPB1 was shown in homozygous SCNT-PSC derived osteoblasts, suggesting a negligible risk of immune rejection of homozygous SCNT PSCs derived lineage cells. Each value is the average of 2 independent experiments (n=3 per experiment).

SUPPLMENTARY EXPERIMENTAL PROCEDURE

Characterization of human SCNT-PSCs

For *in vitro* differentiation assays, SCNT-PSCs were cultured in low-attachment dishes with ESC medium (without bFGF) for 2 weeks in order to obtain embryoid bodies (EBs), and then EBs were analyzed histologically. After fixing, washing, blocking, and permeabilization, EBs were incubated with primary antibodies at 4℃ overnight. After three washes with PBS containing 1% BSA, EBs were stained with secondary antibodies and DAPI for 1 hour and observed under a fluorescence microscope. For teratoma assays, approximately 1x10^5 of undifferentiated SCNT-PSCs were injected into the testicle of a severe combined immunodeficiency (SCID) mouse (Chung et al., 2014). Also, karyotype analysis, genomic DNA fingerprinting, and mitochondrial DNA genotyping of three SCNT-PSC lines were performed using a standard protocol as previously described (Chung et al., 2014).

Differentiation into RPEs

RPE production from SCNT-PSCs (CHA-FT-NT17 and CHA-FT-NT18 as SCNT-PSCs) and ESCs (MA09) was performed using a well-established protocol as previously described (Lu et al., 2009). The pigmented RPE cells were passaged and harvested at passage 3 for characterization. To characterize, SCNT-PSC-derived RPEs were incubated with primary antibodies [anti-PAX6 (Millipore), anti-Bestrophin (Novus), anti-RPE65 (Abcam), anti-Mitf (Millipore), and anti-Sodium-Potassium exchange pump (Abcam) antibodies] diluted 1:100 in blocking solution overnight at 4℃. The cells were then incubated with Alexa Fluor 647-conjugated anti-rabbit IgG (GIBCO) and Alexa Fluor 488-conjugated anti-mouse IgG (GIBCO) for 1 hour at RT. For nuclear staining, cells were incubated with DAPI (Thermo) for 5 minutes; negative controls were incubated with secondary antibodies alone. Stained cells were observed under a Axiovert 200M inverted fluorescence microscope (Carl Zeiss). Flow cytometry analysis was used to measure the expression of RPE-specific markers. To test phagocytosis activity, SCNT-PSC-derived RPEs were treated with a fluorescent-labeled bio-particle™ (Vybrant® Phagocytosis assay kit, Life technologies) according to the manufacturer's instructions. Phagosomes were analyzed by flow cytometry using a FACSCalibur™ (BD). A minimum of 10,000 cells were analyzed for each sample. To confirm the polarized secretion of vascular endothelial growth factor (VEGF) from the basal side of RPE cells and pigment epithelium-derived factor (PEDF) from the apical side of RPE cells, SCNT-PSC-derived RPEs were seeded onto a 6 trans-well culture dish (Corning) and then differentiated to mature RPEs. The media from both the upper and lower reservoirs were collected after 30 days of culture. The amount of secreted proteins was measured by the Quantikine human VEGF ELISA kit (R&D systems) and the human PEDF ELISA kit (Biocompare) according to manufacturer's instruction. All assays were performed in triplicate.

Differentiation into neuronal lineage and characterization

The undifferentiated PSC lines (CHA-hES15 as conventional ESC; CHA-FT-NT17 and CHA-FT-NT18 as SCNT-PSCs) were maintained and differentiated into neuronal lineage as described previously (Cho et al., 2008). Briefly, EBs from human PSC colonies were differentiated to neural precursor cells (NPCs) via neural rosette-like structures and spherical neural masses (SNMs). Most of the SNMs derived from SCNT-PSCs and ESCs expressed NESTIN and SOX2, markers for neural precursor cells (NPCs). Most of the single cell-dissociated NPCs were NESTIN and SOX2 positive, and after 10 days, half of the differentiated cells were TUJ1 positive and the other half GFAP positive (Figure S5). Immunocytochemisty was performed as described previously (Kim et al., 2017). Cells were incubated with primary antibodies at 4°C overnight. The following primary antibodies were used: rabbit polyclonal antibodies, SOX2 (Sigma), NESTIN (BioLegend), tyrosine hydroxylase (TH) (Pel-Freez), neuron-specific class III beta-tubulin (TuJ1) (Covance); mouse monoclonal antibodies, TuJ1 (Covance), GFAP (ICN Biochem), Ki67 (Novocastra), IBA1 (FUJIFILM), MHC Class II (Abcam). MHC Class I (HLA A+HLA B, Abcam).

Differentiation into MPCs and their characterization

PSC-derived MPCs (from CHA-hES15, CHA-FT-NT17, and CHA-FT-NT18) were produced by the method as described previously (Jun et al., 2019). For flow cytometry analysis of cell surface markers, SCNT-PSCs and ESCs-derived MPCs were washed once with DPBS without Ca^{2+}/Mg^{2+} and fixed for 1 hour in a pre-chilled 4% PFA solution (EL Bio) at 4℃. A phycoerythrin (PE)-conjugated mouse anti-human TRA-1-60 (BD) and an allophycocyanine (APC)-conjugated mouse anti-human/mouse SSEA4 (R&D systems) were used as stemness markers; APC-conjugated mouse anti human CD34 (BD) and APC-conjugated mouse anti-human CD45 (BD) were used as hematopoietic markers; APC-conjugated mouse anti-human CD29 (BD), APC-conjugated mouse anti-human CD44 (BD), APC-conjugated mouse anti-human CD90 (BD), and APC-conjugated mouse anti-human CD105 (BD) were used as the MPC markers. After incubation and washing, cells were analyzed using an BD Accuri™ C6 Plus Flow cytometer equipped with the Cell Quest software (BD).

Differentiation into Adipocytes, Osteocytes, and Chondrocytse

For adipogenic differentiation, SCNT-PSCs and ESCs-derived MPCs were seeded onto 0.1% gelatin-coated (Sigma) 12-well tissue culture dish (Nunc) at a concentration of $2x10^5$ cells/well in MPC expansion media. When the cells were fully confluent, the adipogenic differentiation medium (StemPro® Adipogenesis Differentiation Kit, GIBCO) was added to induce adipocyte differentiation (21 days). Cells were stained with an Oil Red O solution (IHC world) according to the manufacturer's instructions. For osteogenic differentiation, osteogenic differentiation media (StemPro® Osteogenesis Differentiation Kit, GIBCO) was added to induce osteocyte differentiation (21 days). Cells were stained with an Alizarin red solution (IHC world) according to the manufacturer's instructions. For chondrogenic differentiation, MPCs were seed in a 15 mL conical tube (BD) at a concentration of $5x10^5$ cells, in chondrogenesis medium (StemPro® Chondrogenesis Differentiation Kit, GIBCO) for approximately 28 days. Differentiated cells were fixed and then embedded in paraffin. The sectioned samples were stained with an Alcian blue staining solution (IHC World) according to the manufacturer's instructions.

RT-PCR or Real-Time PCR of MPC-derived differentiated cells

PCR and real-time PCR were performed on a Bio-Rad thermal cycler (Bio-Rad) or on a Real-time PCR detection system (Bio-Rad). Primers of lineage-specific markers were designed using the Primer Express version 1.0 (PE Applied Biosystems) using RNA sequences obtained from GenBank. Primer sequences are as followed: adipocyte-related genes: *PPARG* (*PPAR-γ*; forward 5'-TGTCTCATAATGCCATCAGGTTTG-3', reverse 5'-GATAACGATGGTGATTTGTCTGTT-3', 224bp) and *CEBPA* (*C/EBP-α*; forward 5'-GCAAACTCACCGCTCCAATG-3', reverse 5'-TTAGGTTCCAAGCCCCAAGTC-3', 247bp), osteocyte-related genes: *RUNX2* (forward 5'-CCCCACGACAACCGCACCAT-3', reverse 5'CACTCCGGCCCACAAATCTC-3', 289bp) and *COL1A1* (*COL1*; forward 5'-AGAACATCACCTACCACTGC-3', reverse 5'-ATGTCCAAAGGTGCAATATC-3', 250bp), and chondrocyte-related genes: *SOX9* (forward 5'-TTCATGAAGATGACCGACGA-3', reverse 5'-CACACCATGAAGGCGTTCAT-3', 326bp), *COMP* (forward 5'-AACGCTGAAGTCACGCTCAC-3', reverse 5'-GGTAGCCAAAGATGAAGCCC-3', 244bp), *Alkaline phosphatase* (forward 5'- ACC ATT CCC ACG TCT TCA CAT TTG -3', reverse 5'- AGA CAT TCT CTC GTT CAC CGC C -3', 250bp), and *OSTEOCALCIN* (forward 5'- ATG AGA GCC CTC ACA CTC CTC -3', reverse 5'- GCC GTA GAA GCG CCG ATA GGC -3', 245bp). *ACTB* (*β-ACTIN*; forward 5'-TGAAGTGTGACGTGGACATC-3', reverse 5'-GGAGGAGCAATGATCTTGAT-3', 152bp)) and *GAPDH* (forward 5'- AGA AGG CTG GGG CTC ATT -3', reverse 5'- AGG GGC CAT CCA CAG TCT -3', 247bp) were used as a loading control.

SUPPLMENTARY REFERENCES

Cho, M.S., Lee, Y.E., Kim, J.Y., Chung, S., Cho, Y.H., Kim, D.S., Kang, S.M., Lee, H., Kim, M.H., Kim, J.H., *et al.* (2008). Highly efficient and large-scale generation of functional dopamine neurons from human embryonic stem cells. Proc Natl Acad Sci U S A *105*, 3392-3397.

Chung, Y.G., Eum, J.H., Lee, J.E., Shim, S.H., Sepilian, V., Hong, S.W., Lee, Y., Treff, N.R., Choi, Y.H., Kimbrel, E.A., *et al.* (2014). Human somatic cell nuclear transfer using adult cells. Cell Stem Cell *14*, 777-780.
Jun, S.M., Park, M., Lee, J.Y., Jung, S., Lee, J.E., Shim, S.H., Song, H., and Lee, D.R. (2019). Single cell-derived clonally expanded mesenchymal progenitor cells from somatic cell nuclear transfer-derived pluripotent stem cells ameliorate the endometrial function in the uterus of a murine model with Asherman's syndrome. Cell Prolif *52*, e12597.
Kim, S.M., Lim, M.S., Lee, E.H., Jung, S.J., Chung, H.Y., Kim, C.H., and Park, C.H. (2017). Efficient Generation of Dopamine Neurons by Synthetic Transcription Factor mRNAs. Mol Ther *25*, 2028-2037.
Lu, B., Malcuit, C., Wang, S., Girman, S., Francis, P., Lemieux, L., Lanza, R., and Lund, R. (2009). Long-term safety and function of RPE from human embryonic stem cells in preclinical models of macular degeneration. Stem Cells *27*, 2126-2135.
