# Supplementary material for: Cryopreserved Human Oocytes and Cord Blood Cells Can Produce Somatic Cell Nuclear Transfer-Derived Pluripotent Stem Cells with a Homozygous HLA Type
Source: Stem Cell Reports. 2020 Jun 4;15(1):171–84. doi: 10.1016/j.stemcr.2020.05.005 (PMC7363744; doi:10.1016/j.stemcr.2020.05.005)
Supplement: Document S2. Article plus Supplemental Information [file mmc2.pdf]

Stem Cell Reports
Article
OPEN ACCESS

Cryopreserved Human Oocytes and Cord Blood Cells Can Produce Somatic Cell Nuclear Transfer-Derived Pluripotent Stem Cells with a Homozygous HLA Type

Jeoung Eun Lee,[1,8] Ji Yoon Lee,[1,8] Chang-Hwan Park,[2,8] Jin Hee Eum,[3] Soo Kyung Jung,[1] A-Reum Han,[4] Dong-Won Seol,[4] Jin Saem Lee,[2] Hyun Soo Shin,[5] Jung Ho Im,[5] Taehoon Chun,[6] Kyungsoo Ha,[7] Deok Rim Heo,[7] Tae Ki Yoon,[4] and Dong Ryul Lee[1,4,*]
[1]CHA Advanced Research Institute, CHA University, Seongnam, Gyunggi-do 13488, Korea
[2]Graduated School of Biomedical Science and Engineering, Hanyang University, Seoul 04763, Korea
[3]Fertility Center of CHA Gangnam Medical Center, CHA University, Seoul 06135, Korea
[4]Department of Biomedical Science, CHA University, Seongnam, Gyunggi-do 13488, Korea
[5]Department of Radiation Oncology, CHA Bundang Medical Center, CHA University, Seongnam 13496, Korea
[6]Department of Biotechnology, College of Life and Biotechnology, Korea University, Seoul 02841, Korea
[7]New Drug Development Center, Osong Medical Innovation Foundation, Osong 28160, Korea
[8]Co-first author
*Correspondence: drleedr@cha.ac.kr
https://doi.org/10.1016/j.stemcr.2020.05.005

SUMMARY

Human pluripotent stem cells (PSCs) through somatic cell nuclear transfer (SCNT) may be an important source for regenerative medicine. The low derivation efficiency of stem cells and the accessibility of human oocytes are the main obstacles to their application. We previously reported that the efficiency of SCNT was increased by overexpression of H3K9me3 demethylase. Here, we applied a modified derivation method to the PSC line and first obtained human SCNT-PSC lines derived from both donated cryopreserved oocytes and cord blood cells with a homozygous human leukocyte antigen (HLA) type. The SCNT-PSCs have very similar characteristics with embryonic stem cells (ESCs) and additionally have shown immunocompatibility in an *in vitro* and *in vivo* humanized mouse with a matching HLA type. Our study demonstrates that SCNT technology using donated cryopreserved oocytes and cord blood cells with a known HLA type provides a promising method for establishing a human HLA-matched SCNT-PSC bank for regenerative medicine.

INTRODUCTION

Somatic cell nuclear transfer (SCNT) mediated by oocytes can produce cloned embryos in more than 20 species (Gurdon, 1962; Kato et al., 1998; Rodriguez-Osorio et al., 2012; Wakayama et al., 1998; Wilmut et al., 1997), and it has contributed to the establishment of pluripotent stem cells (PSCs) in various mammals, including humans (Chung et al., 2014; Tachibana et al., 2013; Yamada et al., 2014). In particular, PSCs may be an extremely important source for immunocompatible cell therapy and may enable a novel approach for regenerative medicine (Lanza et al., 1999). However, despite the value of SCNT-PSCs, several limitations, such as the low derivation efficiency of these stem cells due to poor development of cloned human eggs and the complexity of providing steady sources of human oocytes, make it difficult to apply these cells to cell therapy. Recently, our group evaluated a reprogramming barrier that is mediated by abnormally high activity of histone methylation (H3K9me3) during SCNT and partially overcome by mRNA injection of KDM4A (an H3K9me3 demethylase). In fact, the number of cloned eggs arrested at the eight-cell stage was greatly reduced, and the SCNT-PSC derivation rate also increased to 7.1% per donated mature oocyte (Chung et al., 2015). This technology has recently been applied to non-human primate cloning systems and successfully produced cloned macaque monkeys (Liu et al., 2018). Therefore, it appears that a major hurdle for the application of human SCNT-PSCs as sources for cell therapy has been partially overcome, although further research is still needed for better embryonic development.

The other major issue for the successful application of SCNT is securing ethical and steady sources of human oocytes. Recently, cryopreservation of human oocytes has become an integral part of the human assisted reproductive technology program since *in vitro* fertilization-embryo transfer (IVF-ET) using frozen/thawed oocytes has shown embryonic development and pregnancy rates similar to those achieved using fresh oocytes (Practice Committees of American Society for Reproductive Medicine and Society for Assisted Reproductive Techonology, 2013). Although variations in clinical outcome caused by the quality of frozen/thawed oocytes are still controversial, oocyte cryopreservation has been widely applied for fertility preservation in unmarried and married women. For this reason, cryopreserved human oocytes after the storage period may be a steady source of donor oocytes for SCNT-PSCs, and this

This is an open access article under the CC BY license (http://creativecommons.org/licenses/by/4.0/).

approach could reduce the ethical dilemma caused by unnecessary ovarian hyperstimulation of women for research purposes. However, successful production of cloned embryos using cryopreserved human oocytes and the derivation of SCNT-PSC lines has still not been achieved until now. In a recent animal study, we found that cryopreserved mouse oocyte cytoplasm has a lower potential for SCNT-mediated reprogramming than fresh oocytes, possibly due to increased apoptosis and altered gene expression resulting from cryoinjury (Lee et al., 2019).

It is well known that immune rejection of transplanted cells from recipient targets should be overcome for the clinical application of PSCs in stem cell therapy. Although, autologous PSCs obtained from SCNT or induced PSC (iPSC) technologies can avoid immune rejection by the patient's immune system (Lanza et al., 1999; Mandai et al., 2017), it has been suggested that the use of autologous PSCs is not a good option for patients because it is a less economical and more time-consuming procedure. To overcome these obstacles, research groups have recently suggested another strategy using a homozygous HLA genotype-matched PSC bank that provides stem cells useful to allogeneic users (Lee et al., 2016; Turner et al., 2013). In fact, several reports from the UK and Japan have postulated that 150 and 140 HLA-homozygous iPSCs could match more than 90% of their populations (Okita et al., 2011; Taylor et al., 2012) and a modeling study also suggested that the construction of cell banks of top-ranked haplolines could match a majority of individuals in a multiethnic and admixed population, such as California (Pappas et al., 2015). In addition, the clinical significance of the HLA-homozygous iPSC bank is supported by recent reports showing a lack of T cell response to human iPSC-derived retinal pigment epithelial cells from HLA-homozygous donors and successful transplantation in the major histocompatibility complex (MHC)-matched monkey model (Sugita et al., 2016a, 2016b). Based on these reports, several researchers have started establishing homozygous iPSC lines using fresh blood cells (Rim et al., 2018; Sugita et al., 2016b). Nucleated cells in fresh peripheral and cord blood would be suggested as a noninvasive cell source for the production of iPSCs, but this approach has shown low reprogramming efficiency compared with fibroblasts (Loh et al., 2009). However, despite several successful applications of nuclear donor cells from fresh blood for the production of HLA-homozygous PSCs, a highly labor-intensive process may be required to obtain proper blood cells from blood donors who do not know their HLA information. It was also suggested that frozen cord blood cells stored in a public cell bank could be a useful source to obtain nuclear donor cells with a known HLA type for SCNT, which requires a small number of mononucleated cells (MNCs) for reprogramming.

RESULTS

Derivation of Human SCNT-PSCs Using Cryopreserved Human Oocytes and Its Characterization

To analyze the potential of cryopreserved human oocyte cytoplasm for SCNT-reprogramming, two types of nuclear donor cells were prepared for SCNT. One type was human dermal fibroblast (hDF) cells donated from a 42-year-old female patient with central areolar choroidal dystrophy (center spared, one of eye disease). The other type was MNCs from donated/cryopreserved cord blood with a homozygous human leukocyte antigen (HLA). First, to examine the efficiency of SCNT-mediated reprogramming in frozen/thawed oocytes, we used our recent SCNT protocol using histone demethylase after reconstruction of enucleated oocytes and nuclear donor cells (Chung et al., 2015). A total of 11 frozen/thawed oocytes were enucleated and reconstructed with hDF cells (Figures 1A and 1B). All reconstructed embryos were cleaved, but only three reconstructed embryos (27.3%) developed to the eight-cell stage. One of them was developed further to an expanded blastocyst at 6 days (Figure 1B). This expanded blastocyst was plated onto irradiated mouse embryonic fibroblast (MEF) cells and eventually resulted in a stable human SCNT-PSC line (CHA-FT-NT17, Figure 1B). In the next experiment, a total of 24 frozen/thawed oocytes were also enucleated and then reconstructed by piezo injection of MNCs obtained from frozen/thawed cord blood. Similarly, all the embryos were cleaved, but seven embryos (29.2%) developed to the eight-cell stage. Two of them (8.6%) became expanded blastocysts and then formed two stable human SCNT-PSC lines (CHA-FT-NT18 and CHA-FT-NT19, Figure 1B). When compared with our fresh SCNT, embryonic development from frozen SCNT was poor, but PSC derivation rate was similar to those using fresh oocytes (Figure S1).

As shown in Figures 2 and S2, the morphology of the three SCNT-PSCs was very similar to that of conventional ESCs, and the cells had high alkaline phosphatase activity and high expression of pluripotency marker genes (*POU5-F1*(*OCT4*), *SOX2*, and *NANOG*). In addition, immunostaining revealed that OCT4, SSEA4, TRA-1-60, and TRA-1-81 were expressed in all colonies of the three cell lines (Figure S2). In cytogenetic analysis, the karyotypes of CHA-FT-NT17 derived from hDFs and CHA-FT-NT18 derived from cord blood MNCs were normal (46, XX and 46, XY), while that of CHA-FT-NT19 from the same MNCs was abnormal (47, XYY) (Figure 2A). In addition, molecular genotyping showed that short tandem repeat markers of all SCNT-PSC lines perfectly matched those of their somatic (nuclear) donors (Figure 2B). The mitochondrial genomes of all SCNT-PSC lines were mismatched with those of their somatic (nuclear) donors (Figure 2C), which may suggest

A

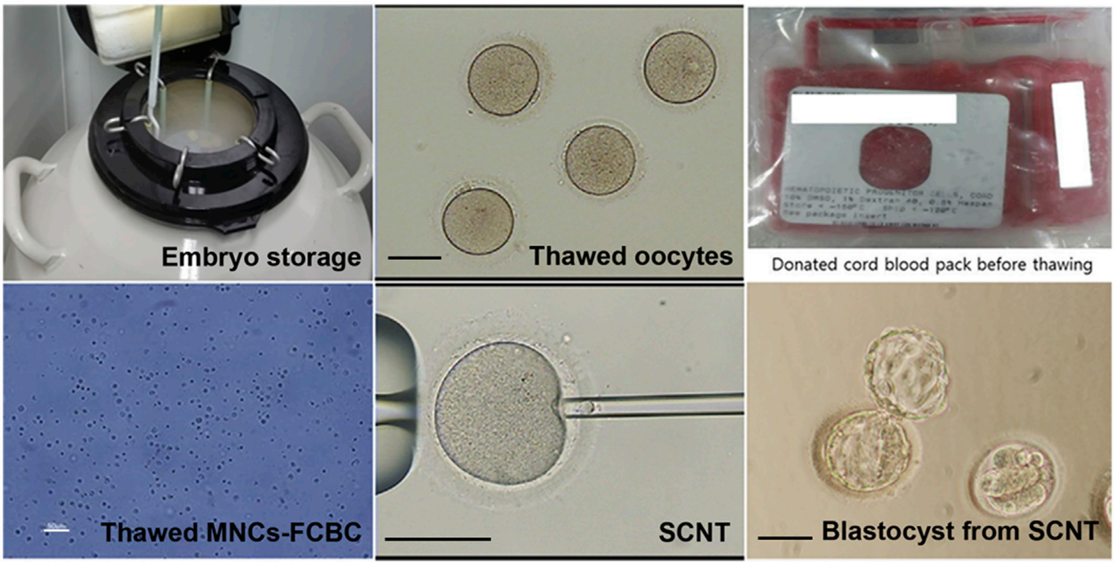

B

Groups	No. of donated MII oocytes	No. of reconstructed oocytes (%)	No. of cleaved (%)	No. of 8-cell (%)	No. of morula (%)	No. of blastocysts (%)	No. of expended blastocysts (%)	No. of SCNT-PSC derived (%)
hDF	11	11 (100)	11 (100)	3 (27.3)	1 (9.1)	1 (9.1)	1 (9.1)	1 (9.1)
MNCs-FCBC	24	24 (100)	16 (66.7)	7 (29.2)	5 (20.8)	4 (16.7)	2 (8.3)	2* (8.3)
Total	35	35 (100)	27 (77.1)	10 (28.6)	6 (17.1)	5 (14.3)	3 (8.6)	3 (8.6)

hDF: human dermal fibroblasts
MNC-FCBC: mononucleated cells from frozen-thawed cord blood cells
SCNT: somatic cell nuclear transfer
*One is normal karyotype and the other is aneuploidy (46, XY and 47, XXY)

Figure 1. Human SCNT
(A) Photographs of the SCNT procedure using cryopreserved oocytes and MNCs from fetal cord blood cells (FCBCs), and embryonic results from SCNT experiments. Scale bars, 100 μm.
(B) Embryonic results from human SCNT experiment.
See also Figure S1.

that their cytoplasm originated from donated cryopreserved oocytes. However, we did not confirm matching to the mitochondrial genotype of oocyte donors because we cannot secure cumulus cells for mitochondrial DNA genotyping from cryopreserved oocytes, which had been almost denuded before vitrification. Other characteristics of pluripotency, such as formation of three-germ layers *in vitro* and *in vivo*, are shown in Figure S2. These results clearly indicate that newly formed CHA-FT-NT17, CHA-FT-NT18, and CHA-FT-NT19 are reprogrammed PSC lines.

Differentiation into Various Lineages Cells

To analyze the possibility of using the newly established SCNT-PSCs as sources for cell therapy, two normal SCNT-PSC lines from cryopreserved oocytes (CHA-FT-NT17 and CHA-FT-NT18) were differentiated into ectoderm lineage cells, retinal pigmented epithelial cells (RPEs), and neuronal progenitor cells (NPCs), using well-established protocols (Cho et al., 2008; Schwartz et al., 2012; Song et al., 2015; Zhang et al., 2001), and then compared with well-characterized ESC lines (MA09 and CHA-hES-15, respectively)

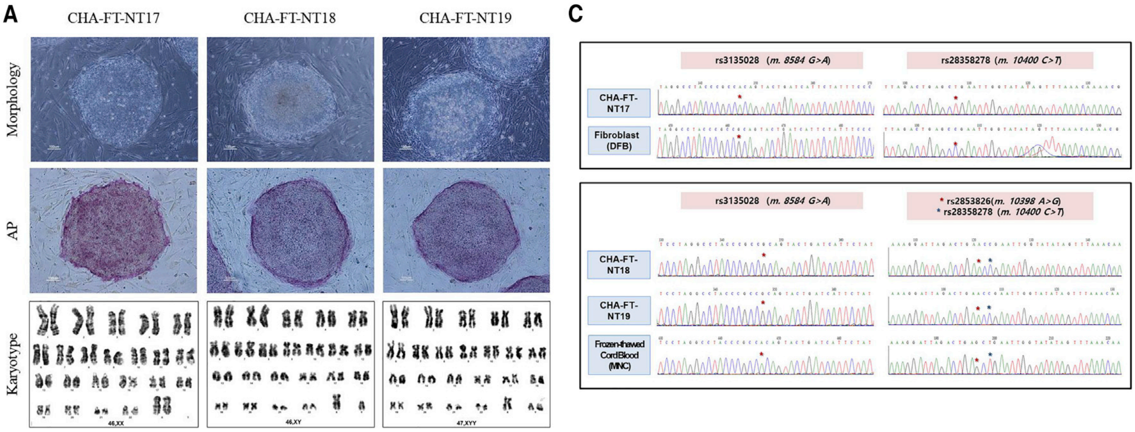

B

	Somatic donor (hDF)		CHA-FT- NT17		Somatic donor (Cord blood MNC)		CHA-FT- NT18		CHA-FT- NT19	
D8S1179	11	13	11	13	10	15	10	15	10	15
D21S11	30	31	30	31	29	30	29	30	29	30
D3S1358	16	17	16	17	15	17	15	17	15	17
THO1	6	10	6	10	9	9.3	9	9.3	9	9.3
D19S433	13	15	13	15	13	14	13	14	13	14
vWA	17	18	17	18	17	18	17	18	17	18
AMEL	X	X	X	X	X	Y	X	Y	X	Y
FGA	19	25	19	25	20	24	20	24	20	24

Figure 2. Characterization of Human SCNT-PSC Lines

(A) Morphology, alkaline phosphatase (AP) staining, and karyotypes of SCNT-PSC lines from cryopreserved oocytes (CHA-FT-NT17, CHA-FT-NT18, and CHA-FT-NT19). Three PSC lines showed embryonic stem cell-like morphology and positive signals for AP. Cytogenetic G-banding analysis showed that CHA-FT-NT17 and CHA-FT-NT18 had normal karyotypes (46, XX and 46, XY, respectively), but CHA-FT-NT19 had an abnormal karyotype (47, XYY). Scale bars, 100 μm.

(B) Mitochondrial (mt) DNA genotyping of three single nucleotide polymorphic sites. m.8584 G > A (rs3135028), m.10400C > T (rs28358278), m.10398A > G (rs2853826), and m.10400C > T (rs28358278) demonstrate that the mtDNAs in the three SCNT-PSC lines were derived from the oocytes, not hDFs or cord blood MNCs.

(C) Nuclear DNA genotyping using eight short tandem repeat markers (seven at autosomal loci and one at the X/Y locus) showed that the nuclear DNAs in three SCNT-PSC lines were exclusively derived from somatic donor cells (hDFs or cord blood MNCs).

See also Figure S2.

(Figures S3 and S4). In the characterization of PSC-derived RPEs and NPCs, we did not find any difference in the differentiation capability between SCNT-PSCs derived from cryopreserved oocytes as a cytoplasm source and cryopreserved cord blood cells as nuclear donors for the reprogramming procedure. Generated NPCs successfully expressed MHC class I and II antigens, implying maturation (Figure S5).

Next, we explored whether CHA-FT-NT17 and CHA-FT-NT18 cells were also differentiated into mesoderm lineage cells, such as mesenchymal progenitor cells (MPCs) and osteoblasts, by using a previously established protocol (Jun et al., 2019). Clonally expanded MPCs displayed traditional markers for MPCs, such as the CD29^{+}, CD44^{+}, CD90^{+}, CD105^{+}, CD45^{-}, and CD34^{-} phenotypes and potential capacity for differentiation into adipocytes, osteocytes, and chondrocytes under differentiation conditions. In addition, the precursor genes for multilineage differentiated cells, *CEBPA* (*C/EBPα*), *RUNX2*, and *SOX9*, were clearly expressed in both CHA-FT-NT17- and CHA-FT-NT18-derived cells. The maturation-related genes *PPARG* (*PPARγ*, adipocytes), *COL1A1* (*COL1*, osteocytes), and *COMP* (chondrocytes) were detected after differentiation. In addition, we measured the cumulative cell number of both SCNT-PSC-MPCs with different sources of donor cells and found that both cells were highly increased until 18 passages (Figure S6). These results may show that homozygous SCNT-

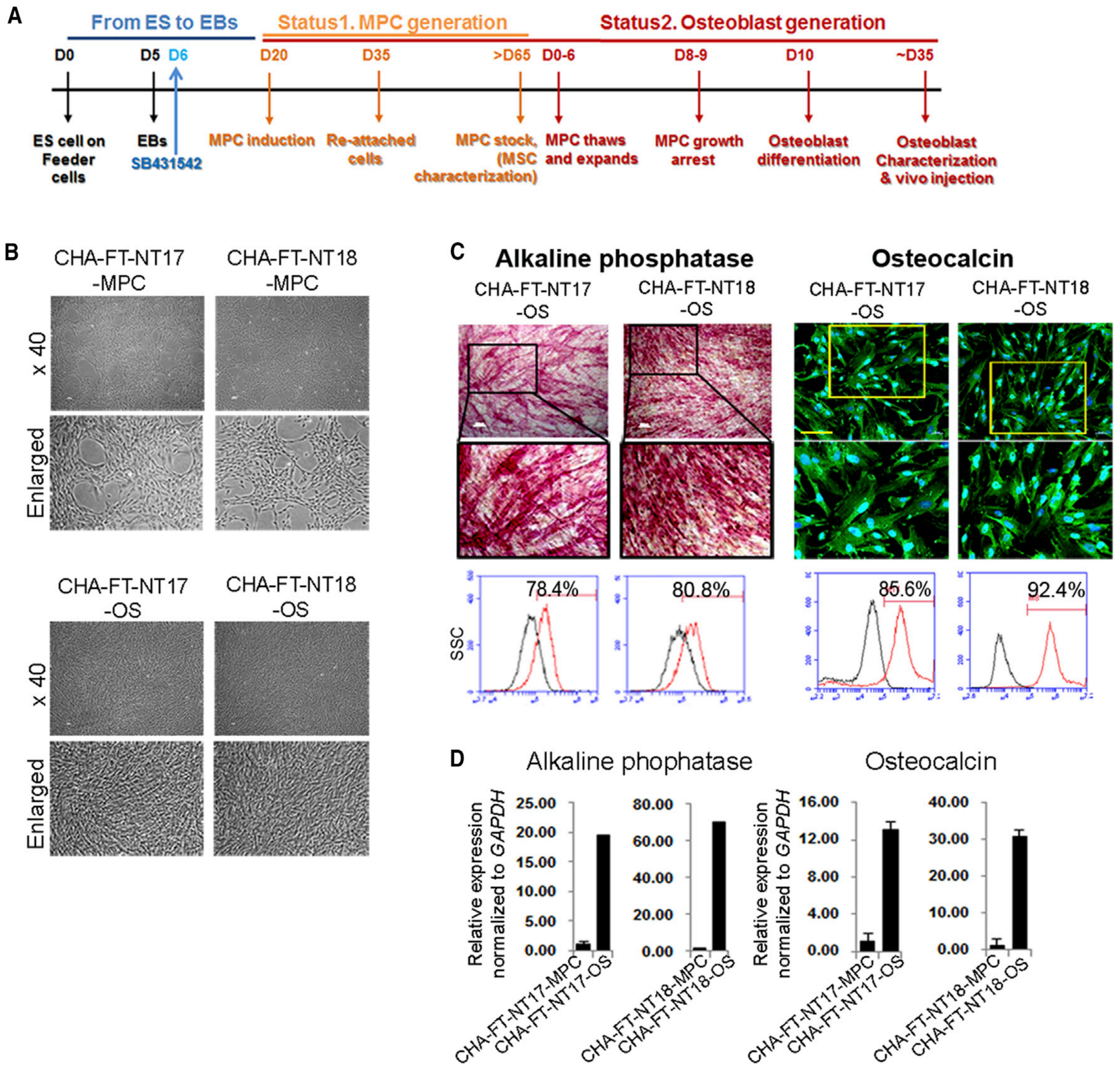

Figure 3. Production of MPCs and Osteoblasts from Human SCNT-PSCs with a Homozygous HLA Type

(A) Schematic diagram showing the experimental procedure *in vitro*.

(B) The representative morphology of SCNT-PSC (CHA-FT-NT17 and CHA-FT-NT18)-derived MPCs and osteoblasts (OS). Scale bars, 100 μm (×40).

(C and D) SCNT-PSC-OS were characterized by high alkaline phosphatase activity and osteocalcin expression. ALP activity and osteocalcin expression of SCNT-PSC-OS showed by ALP staining and immunocytochemistry (C, upper panel), FACS (C, lower panel), and quantitative RT-PCR (D). Scale bars, 100 μm (white) and 20 μm (yellow).

See also Figures S3 and S4.

PSCs derived using cryopreserved oocytes (CHA-FT-NT18) can be successfully differentiated into mesodermal and ectodermal lineage cells.

Immunocompatibility of Homozygous SCNT-PSC-Derived Somatic Cells *In Vitro*

To investigate the immune rejection response of SCNT-PSC-derived differentiated cells, allogeneic cells with different levels of MHC expression are needed. Osteoblasts from both SCNT-PSC-MPCs presented positive staining for alizarin red, osteocalcin, and alkaline phosphatase, suggesting differentiation into mature osteoblasts (Figures 3A–3D). In fact, SCNT-PSC-MPCs showed low expression of MHC class II, but further differentiated SCNT-PSC-osteoblasts expressed high levels of MHC class II molecules (Figure 4A). To analyze the immunocompetence of those cells, T cell activation against CHA-FT-NT18 was monitored after coculture with HLA-matched or HLA-mismatched $CD4^+$

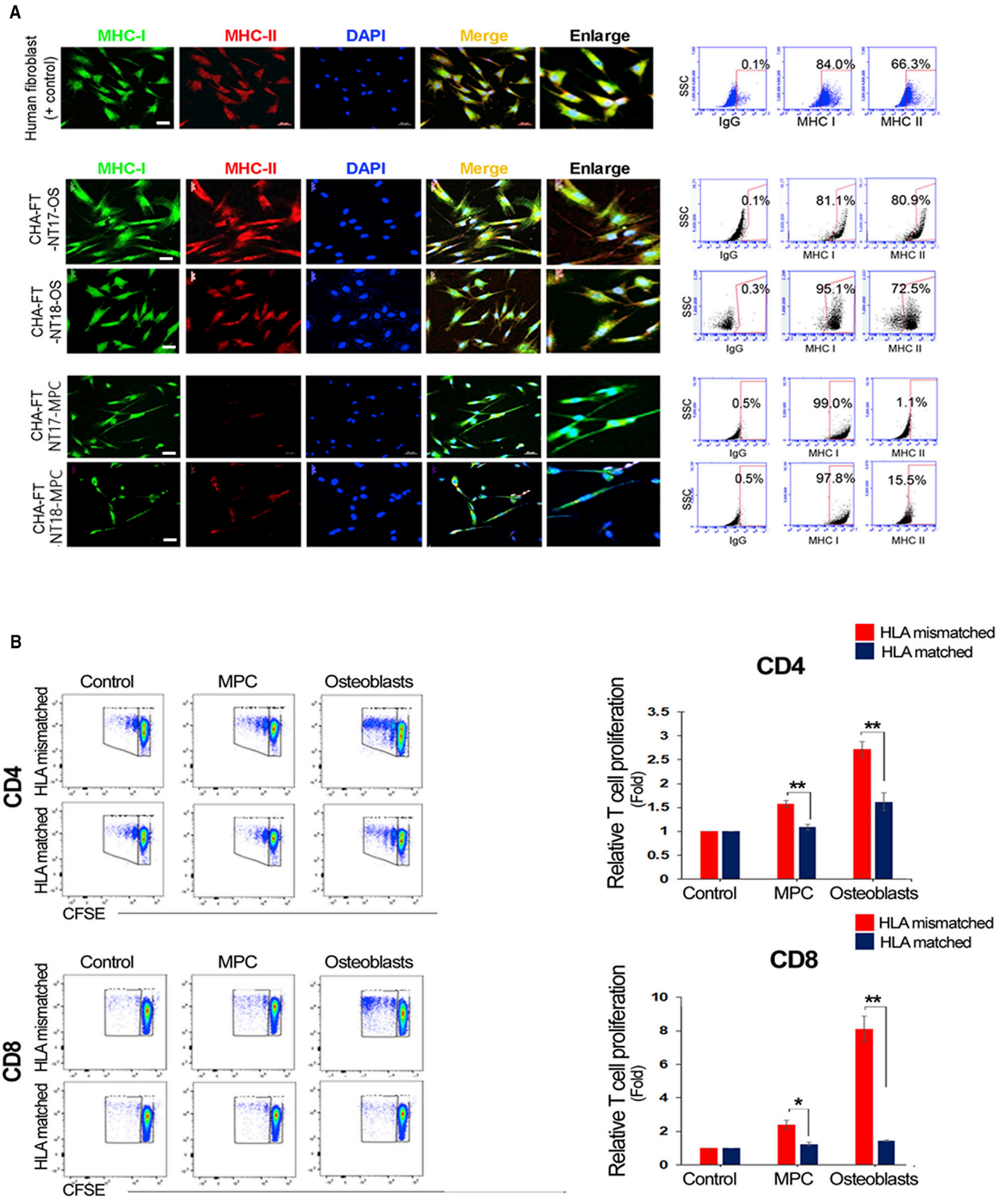

(legend on next page)

and CD8+ T cells. CHA-FT-NT18-derived somatic cells (MPCs and osteoblasts) can induce the proliferation of T cells according to the HLA allele. In this study, regardless of lineage cell type, the proliferation of HLA-matched T cells against homozygous CHA-FT-NT18 type (HLA-A:33:03,33:03; HLA-B:44:03,44:03; HLA-DRB1:13:02,13:02)-derived somatic cells was significantly lower than that of HLA-mismatched T cells against a heterozygous CHA-FT-NT17 type (HLA-A:01:01,02:01; HLA-B:35:01,37:01; HLA-DRB1:10:01,14:54)-derived somatic cells (HLA mismatched versus HLA matched; in MPCs, CD4, 1.5- ± 0.4-fold, CD8, 2.5- ± 0.3-fold; in osteoblasts, CD4, 1.8- ± 0.7-fold, CD8, 8.3- ± 1.3-fold) (Figure 4B). To further examine the immunogenicity by minor HLA-II, CD4 T cell proliferation was performed using mismatched group for HLA-II with HLA-I matching against SCNT-osteoblasts. First, we selected peripheral blood monocytes (PBMCs) having matched HLA-I (A, B, and C) and HLA-II (DR, DQ, and DP), which is referred to as a matched group, and having mismatched DP type of HLA-II, which is referred to as a mismatched group. This mismatched group means all matched HLA types except HLA-II DP. DNA sequencing data in this study are listed in Figure S7A. As expected, no significant difference was found by minor HLA-II DPB1 in SCNT-derived osteoblasts and it is similar to relative T cell proliferation of negative control, suggesting a negligible risk of immune rejection in therapeutic treatment (Figure S7B). Although osteoblasts having strong immune response displayed no significant difference by minor HLA DPB1 in immune rejection, we cannot still rule out that other alleles or DPB1 from HLA-II of lineage cells may occur strong immune rejection, such as graft-versus-host disease (GVHD) (Morishima et al., 2018). Consistent with previous literature (Chidgey et al., 2008), we found that SCNT cells have very low reaction with no significance in immune rejection, even minor HLA histocompatibility, suggesting the immunocompatibility of homozygous SCNT-PSC-derived somatic cells.

Immunocompatibility of Homozygous SCNT-PSC-Derived Somatic Cells *In Vivo* Humanized Mice

We next sought to examine whether host immune cells according to HLA types were affected by homozygous SCNT-PSC-derived somatic cells *in vivo*. To this end, we established a humanized mouse model that possesses human hematopoietic cells. Magnetic-activated cell sorting (MACS)-sorted cord blood-derived CD34+ (CB CD34+) cells were injected into irradiated 3-week-old NSG (NOD.Cg-Prkdcscid Il2rgtm1Wjl [NSG/SzJ]) mice (Figure 5). The HLA-matched group indicates that HLA-matched T cells reacted against homozygous CHA-FT-NT18-derived osteoblasts in humanized mice and displayed self-signals with all matched HLA-A, B, and DR alleles. In addition, the HLA-mismatched group is the group that has HLA-mismatched T cells against CHA-FT-NT18-derived osteoblasts; ultimately, it was defined as a non-self-signal when HLA-A, B, and DR were at least 75% mismatched.

The schematic plan for animal experiments is shown in Figure 6A. We first confirmed that CD45+ cells in the peripheral blood (PB) were similarly displayed over 20% at 8 weeks after CB CD34+ cell injection for both groups (humanized mice model for HLA-matched cells, 21.2% ± 9.8%; humanized mice model for HLA-mismatched cells, 20.8% ± 4.3%), suggesting no bias in human cell engraftment (Figure 6B). To investigate whether CD3+ lymphocytes from humanized mice were expanded by the recognition of SCNT-PSC-derived osteoblasts, we carried out fluorescence-activated cell sorting (FACS) analysis and found that homozygous CHA-FT-NT18-derived osteoblasts in the HLA-matched group had a weaker effect on the proliferation of mature T cells in the thymus than did those in the HLA-mismatched group. The same CHA-FT-NT18-derived osteoblasts in the HLA-mismatched group could significantly induce increased mature CD4+ lymphocytes at 15 days, mature CD8+ cells at 8 days, and thymocyte precursor CD4+CD8+ cells at 7 days (Figure 6C), suggesting effective suppression of homozygous CHA-FT-NT18-derived cells with HLA matching during the generation of T cells (Figure 6C). To further address the suppressive effects of homozygous CHA-FT-NT18-derived osteoblasts on T cell generation from other organs, humanized mice received a secondary osteoblast injection and were sacrificed at 4 weeks after cell transplantation. These data showed that homozygous SCNT-PSC-derived osteoblasts with HLA-mismatched alleles did not overall increase human immune cells in various

Figure 4. Immunological Characterization of MPCs and Osteoblasts from Human SCNT-PSCs with a Homozygous HLA Type

(A) Expression of MHC antigens by differentiation stage in human PSCs. MHC class I and II antigens were highly expressed in human fibroblasts used as controls. In SCNT-PSC-derived MPC, MHC class I also expressed, but MHC class II was not detected. However, in SCNT-PSC-derived osteoblasts (OS), both MHC class I and II antigens were highly expressed regardless of the cell line. Scale bars, 50 μm.

(B) When HLA-matched CD4+ and CD8+ T cells against homozygous CHA-FT-NT18-derived somatic cells were cocultured, the proliferation of T cells was not increased regardless of the lineage cell type ($p > 0.05$). However, HLA-mismatched CD4+ and CD8+ T cells were dramatically increased by osteoblasts highly expressing MHC molecules in a proliferation assay compared with MPC with relatively low expression of MHC molecules (*$p < 0.05$, **$p < 0.01$).

See also Figure S6.

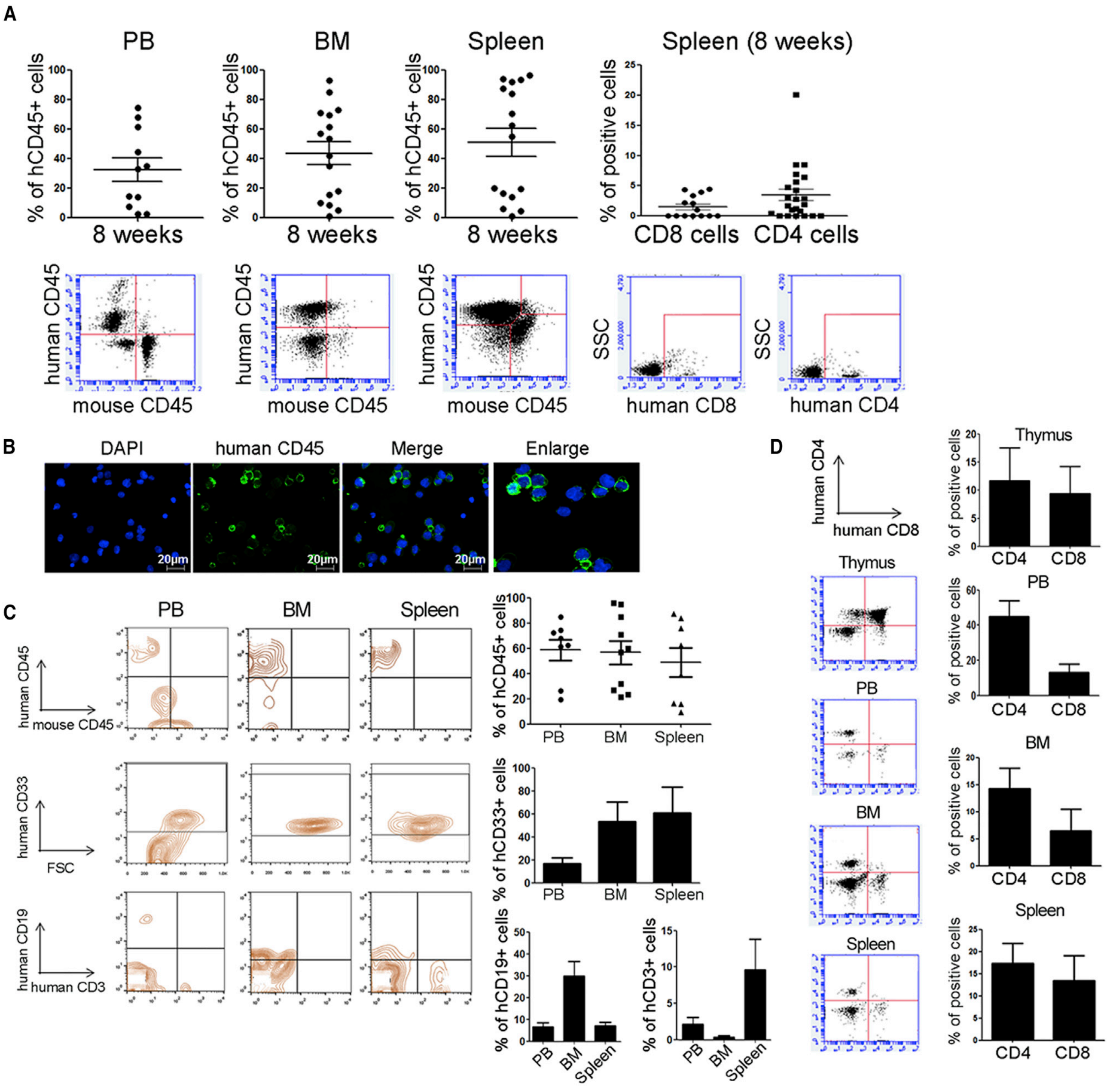

Figure 5. The Establishment of a Humanized Mouse Model Was Confirmed by a Human Pan Hematopoietic Cell Markers

(A) Although the frequencies of T lymphocytes were low, stable engraftment of human cells was revealed in tissues, including the PB, BM, and spleen.

(B) Immunocytochemistry showed that CD45 was detected in the PB. Scale bars, 20 μm.

(C) High engraftment of $CD33^+$ myeloid lineage cells, compared with that of $CD19^+$ and $CD3^+$ lymphoid lineage cells, was revealed by FACS analysis.

(D) In T cells, $CD4^+CD8^+$ immature thymocyte precursors were regenerated in the mouse thymus and mature T cells, such as single $CD4^+$ and $CD8^+$ cells were detected in the PB, BM, and spleen at 18 weeks after transplantation of $CD34^+$ cells.

organs from humanized mice. In particular, $CD4^+$ in the PB, $CD19^+$ in the spleen and bone marrow (BM), and thymocyte precursors in the thymus were significantly higher in the HLA-mismatched group than in the HLA-matched group. T cell receptor αβ (TCRαβ) was highly expressed in $CD4^+$ and $CD8^+$ T cells from the HLA-mismatched group, implying a role for SCNT-PSC-derived osteoblasts in the maturation of immune cells and

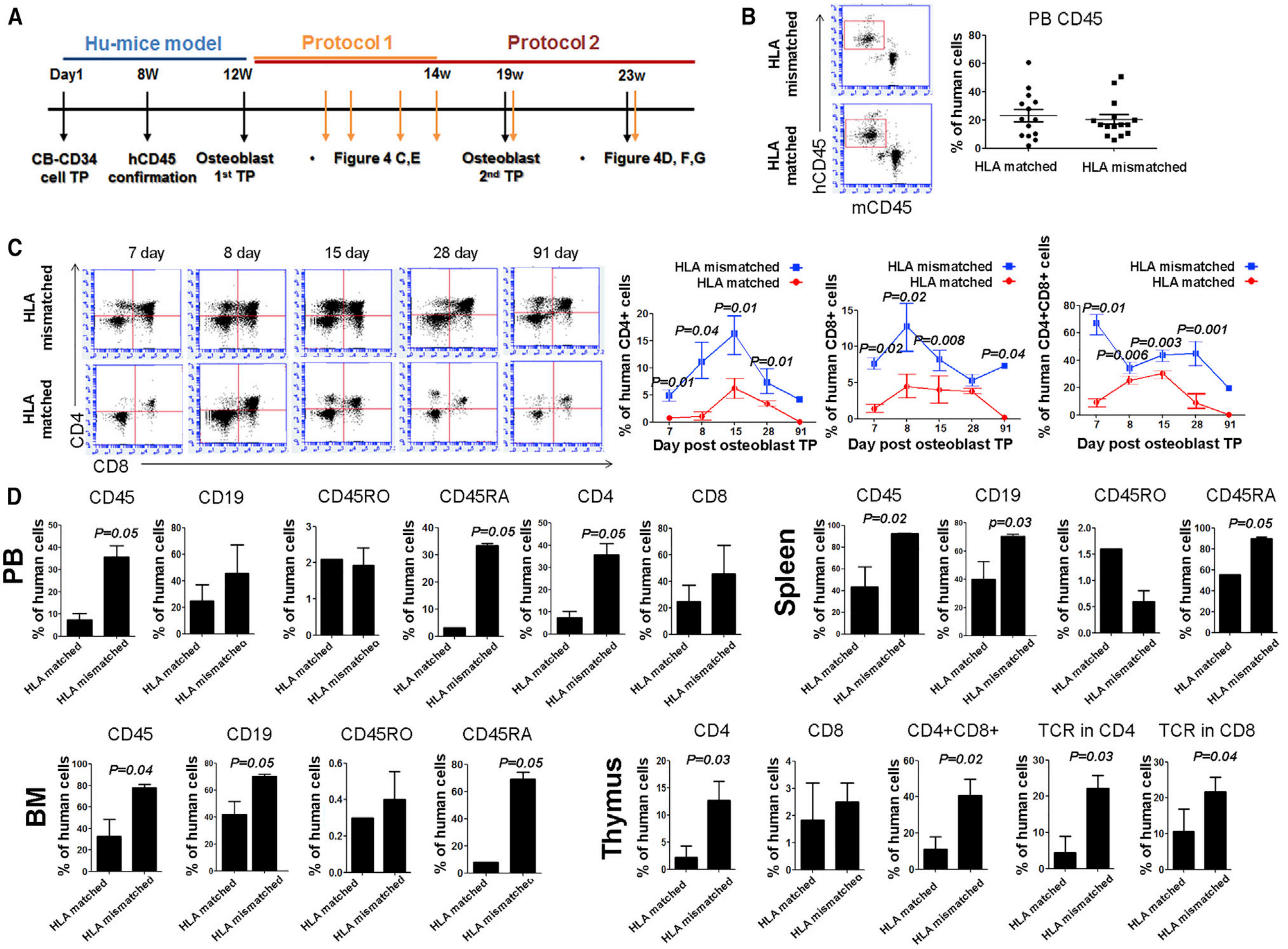

Figure 6. Immunological Characterization of Human SCNT-PSCs with a Homozygous HLA Type *In Vivo* (I)
(A) Schematic diagram showing the plan for animal studies using a humanized mouse model.
(B) FACS analysis showed the frequency of $CD45^{+}$ human cells in a humanized mouse model at 8 weeks after CB $CD34^{+}$ cells. To inject HLA-mismatched and HLA-matched osteoblasts with no bias, humanized mice with the $CD45^{+}$ phenotype were equally divided into two groups.
(C) The thymus from each humanized mouse was collected at 7, 8, 15, 28, and 91 days and then subjected to FACS analysis. FACS analysis showed that CD3 lymphocytes in the thymus were significantly expanded by mismatched human SCNT-PSC-derived osteoblasts compared with matched osteoblasts.
(D) At 23 weeks, hematopoietic markers, including CD45, CD45RA, CD19, and TCR were overall increased in humanized tissues after two injections of mismatched osteoblasts. All results are presented as the means ± SEM.
See also Figures S6 and S7.

reconstitution of the immune network. However, most human $CD45^{+}$ cells were the naive phenotype, CD45RA rather than CD45RO (Figure 6D).

Next, to assess the graft survival of SCNT-PSC-derived osteoblasts *in vivo*, Beriplast-containing SCNT-PSC-derived osteoblasts were injected into the intradermal skin layer. To track the engrafted cells, skin tissues were harvested at 7 and 15 days after cell transplantation and were subjected to immunohistochemistry (IHC). Cells double-stained for human-specific markers (STEM121, Takara) and osteocalcin were counted in the skin. Robust engraftment of CHA-FT-NT18-derived osteoblasts was observed at 3 days (data not shown) and gradually decreased over 7–15 days. Against CHA-FT-NT18, the HLA-mismatched group displayed rapidly disappearing engraftment compared with that of the HLA-matched group (HLA matched versus HLA mismatched at 7 days, 2.33-fold; HLA matched versus HLA mismatched at 15 days, 1.88-fold) (Figure 7A). Intriguingly, immune CD3 T cells were sustained over 15 days, even after the grafted cells disappeared (Figures 6C and 7A). These data suggest that, even without having many remaining engrafted cells, the activity of human immune

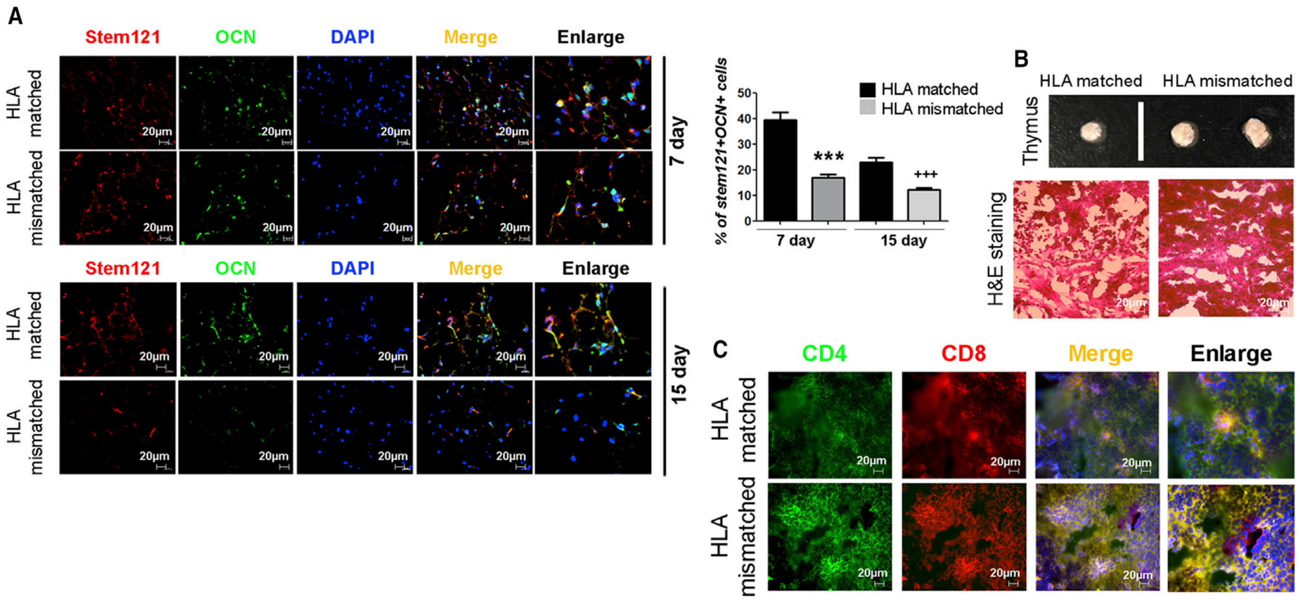

Figure 7. Immunological Characterization of Human SCNT-PSCs with a Homozygous HLA Type *In Vivo* (II)

(A) Engrafted HLA-mismatched human osteoblasts rapidly disappeared. These data are presented as the means ± SEM from at least three experiments. Asterisks depict statistically significant differences compared with HLA-matched human osteoblasts (***$p < 0.001$ at 7 days, $^{+++}p < 0.001$ at 15 days).

(B) Gross examination and H&E staining of the humanized thymus.

(C) Immunocytochemistry also showed that $CD4^+$ and $CD8^+$ cells were qualitatively increased in the thymus with mismatched osteoblasts. Scale bars, 20 μm.

cells can still be sustained in the HLA-mismatched group. To confirm whether the infiltrated cells in the thymus were human $CD3^+$ T cells generated via encouragement from human SCNT-PSC-derived osteoblasts, IHC was next carried out on the thymus with human-specific antibodies for $CD4^+$ and $CD8^+$ at 11 weeks after osteoblast transplantation. Gross examination and H&E staining results showed a larger thymus size and a higher frequency of thymocytes in the HLA-mismatched groups than in the HLA-matched group (Figure 7B). In addition, the data clearly showed a high level of human $CD4^+CD8^+$ thymocytes in humanized mice in the HLA-mismatched group (Figure 7C). From these data, we suggest that homozygous SCNT-PSC-derived osteoblasts could play a role as functional therapeutic cells without stimulating T cell generation.

DISCUSSION

In this study, we have successfully established a human normal SCNT-PSC line with homozygous HLA type, which is derived from both donated cryopreserved oocytes and cord blood cells. At first, to examine the efficiency of frozen/thawed oocytes for SCNT-PSC production, we analyzed the SCNT results indirectly in comparison with our previous results obtained using fresh human oocytes (Chung et al., 2015). Although there was no difference in reconstruction or first cleavage after activation and KDM4A mRNA, the developmental rate after the eight-cell stage and the blastocyst formation rate were substantially decreased in this study using frozen/thawed oocytes. To increase the efficiency of PSC derivation , we applied a novel derivation medium (1:1 mixture of embryonic stem cell [ESC] derivation medium and ESC culture-conditioned medium) for the outgrowth of attached SCNT embryos. Remarkably, all three expanded blastocysts attached to MEFs were outgrown and established stable cell lines (Figures 1B and S1). These results suggest that frozen/thawed human oocytes could have a lower potency for reprogramming the donor nucleus than fresh oocytes, but reprogramming of the donor nucleus mediated by frozen/thawed oocytes can fully support further embryonic development. In addition, in this study, we suggest that a refined culture technique can contribute to increasing the efficiency of PSC derivation from SCNT embryos showing poor development.

Among many features of MPCs, a notable advantage of allogeneic MPCs is evasion from allorejection. Most studies have described human MPCs with MHC class I and rare

MHC class II by showing evidence from *in vitro* experiments (Ryan et al., 2005). Although this evidence cannot fully explain the machinery for the evasion of alloreactivity in MPCs, low expression of MHC molecules in MPCs has hypoimmunogenic properties, which make MPCs promising therapeutic cell sources in regenerative medicine. As expected, HLA-mismatched $CD4^+$ and $CD8^+$ T cells were dramatically increased by osteoblasts with high expression of MHC molecules in a proliferation assay compared with MPCs with relatively low expression of MHC molecules. Because the response to immune rejection is affected by the high expression of MHC molecules in differentiated cells, the use of cell sources with matched HLA types could increase the efficiency of cell transplantation. In addition, SCNT-PSC-derived MPCs have a moderating effect on proliferating T cells when HLA-mismatched $CD4^+$ and $CD8^+$ T cells react, although this effect could induce significantly lower proliferation of T cells than more differentiated types of cells (SCNT-PSC-osteoblasts). This also suggests that a successful hypoimmunogenic response would be achieved by homozygous SCNT-PSC-derived somatic cells (even including MPCs) with matched HLA types. From these results, we propose that the hypoimmunogenic properties of homozygous SCNT-PSC-derived somatic cells will facilitate their use as cell therapeutic sources in regenerative medicine due to safety related to immune conflicts.

In this study, differentiated functional cells from SCNT-PSCs with homozygous HLA type have shown immunocompatibility in humanized mouse with a matching HLA type. However, after secondary osteoblast injection, heterozygous SCNT-PSC-derived osteoblasts did not overall increase human immune cells and produce fully mature immune cells in various organs from humanized mice (Figure 6D). In fact, the thymus is an important organ that produces lymphocytes via positive and negative selection of thymocyte precursors (Cohen et al., 2006). Thymic epithelial cells in the medulla express MHC class II, which can encourage the maturation of thymocytes; however, thymic epithelial cells in a humanized mouse model are mouse, not human, and may lead to the failure of full maturation in lymphocytes. Although several limitations of the humanized mouse, such as improper education of T cells in the murine thymus and inability of the reconstructed system to response against minor histocompatibility-mismatched cells still exist (Kooreman et al., 2017), immune response for major histocompatibility of homozygous SCNT-PSC-derived cells in our study was partly shown by showing a proliferation of human T cells against human cells with mismatched HLA in murine thymus (Figures 6C, 6D, and 7A–7C). We still cannot exclude the possibility that investigation for full immunity against entire human HLA failed due to immaturity of innate immune cells. To overcome this obstacle, further efforts to obtain an advanced humanized mouse model with a functional innate immune system will be required to generate functional T cells (Shultz et al., 2010).

Here, we have reported the successful production of HLA-homozygous SCNT-PSCs using MNCs from frozen/thawed donated cord blood in a public bank and frozen (vitrified) human oocytes donated after storage for 5 years. The homozygous PSCs are produced by frozen oocyte-mediated reprogramming and do not undergo an artificial genetic modulation. However, they have a differentiation capability very similar to that of conventional ESCs and SCNT-PSCs obtained from fibroblasts and/or fresh oocytes. In addition, homozygous SCNT-PSC-derived cells are highly immunocompatible with allogeneic HLA-matched immune cells. Therefore, we may suggest that this SCNT technology facilitates the establishment of the human HLA-matched SCNT-PSC bank.

EXPERIMENTAL PROCEDURES

Ethics Statements

To confirm the potential of cryopreserved human oocytes on SCNT reprogramming, we performed the experimental protocol under approval by both the institutional review board (IRB) of CHA University (1044308-201511-SR-024-02) and the National Institutional Review Board of the Korean Government (no. 3 for SCNT study). Frozen oocytes were donated from the Fertility Center of CHA Gangnam Medical Center under informed consent of patients after 5 years of storing (by the law of the Bioethics and Safety of Republic of Korea). Also, the protocol for recruitment of skin cells was approved by the IRB of CHA Gangnam Medical Center (GCI 13-033) and by the IRB of Bundang CHA General Hospital (protocol 2013-07-077). Frozen cord blood cells were donated from the Public Cord Blood Bank of Bundang CHA General Hospital after the IRB of CHA Gangnam Medical Center approved the protocol for recruitment of donated frozen cord bloods (protocols GCI-13-032).

All animal protocols were reviewed and approved by the Institutional Animal Care and Use Committee of CHA University and all animal procedures were performed in accordance with approved guidelines and regulations. Donations of cord blood used for humanized mice study were approved by the IRB of CHA University (protocols 1044308-201702-BR-017-03, 04) and were obtained from the Public Cord Blood Bank of Bundang CHA General Hospital, Seongnam, Korea.

Preparation of Human Cryopreserved Oocytes

Ovarian stimulation for the oocytes was carried out using a commonly used clinical IVF-ET protocol. Most of the collected cumulus-oocytes complexes were used for conventional IVF-ET

and the remaining were cryopreserved using a vitrification method until further use (Yoon et al., 2007).

Preparation of Fibroblasts, MNCs, and SCNT Using Cryopreserved Oocytes

hDF cells were obtained from an age-related macular degeneration patient. Small pieces of abdominal skin (0.5 × 0.3 cm) were biopsied under local anesthesia and cell preparation was performed as described previously (Chung et al., 2014). Human MNCs were prepared for another SCNT experiment using donated frozen/thawed cord blood with known HLA information for public use (with homozygous allele in HLA-A, B, and DRB1 loci). The MNCs from frozen cord blood were isolated using Ficoll-Paque PLUS (GE Healthcare) after thawing according to the manufacturer's instructions and cultured *in vitro* before use. The procedure of SCNT was performed by using our protocol as described previously (Chung et al., 2014; Matoba et al., 2014).

Derivation of Human SCNT-PSCs from Cloned Blastocysts

After removal of the zona pellucida using an acidic Tyrode solution (Merck), whole blastocysts were plated onto MEFs in a mixed medium (1:1 ratio): the conditioned hESC culture medium and the original hESC derivation medium (DMEM/F12 supplemented with 20% KnockOut Serum Replacement [KSR] [Gbico], 20 ng/mL human recombinant basic fibroblast growth factor [bFGF] [Giboc], and 2,000 units/mL recombinant human leukemia inhibitory factor [Giboc]). The new derivation medium was not changed for the next 3 days and then half of the media volume was replaced with fresh medium daily. After the second passage, PSC cells were maintained in DMEM/F12 medium supplemented with 20% KSR, 1% (v/v) non-essential amino acids (Gibco), 0.1mM β-mercaptoethanol (Gibco), and 4 ng/mL bFGF.

Expression of HLA Subtypes

To determine the expression of HLA subtypes on SCNT-PSC- and ESC-derived MPCs, cells were washed and fixed. After permeabilization, washing, and blocking, cells were incubated with primary antibodies (anti-human MHC class I [HLA-A + HLA-B, Abcam] and anti-human MHC class II [HLA DO, Abcam]) for 2 h at room temperature.

In Vitro T Cell Proliferation Assay Using SCNT-PSC-Derived MPCs and Osteocytes according to HLA Matching

Human PBMCs were isolated from blood samples obtained from healthy donors, and their HLA types were analyzed by the hematopoietic stem cell bank at the Catholic University, Seoul, Korea. PBMCs displaying HLA types equal to those in SCNT-PSC (CHA-FT-NT18)-derived cells were used for this study. In brief, PBMCs were maintained in AIM-V medium (Gibco) supplemented with DNase overnight; the next day, cells were harvested and incubated with carboxyfluorescein diacetate succinimidyl ester (CFSE) (Thermo Fisher Scientific) for 10 min. CFSE-incorporated PBMCs were plated in 24-well plates at a density of 1×10^6 cells/well. The test cells were harvested, washed three times with AIM-V medium, and cocultured with PBMCs. For the control, PBMCs were treated only with the AIM-V medium (without test cells). After a 7-day coculture, fresh test cells were added to the PBMCs for a second stimulation and cultured for an additional 7 days. Then, cells were harvested and stained with CD4 (BD) and CD8 (BD) antibodies to then measure T cell proliferation using FACSCanto II. To investigate immune rejection by mismatched HLA-II DPB1, full mismatched cells with cytokine were used as a positive control against CHA-FT-NT18 cells.

Establishment of Humanized Mice and *In Vivo* Assay

To establish a humanized mouse model, human CB CD34$^+$ cells were isolated by MACS sorting; their purity after sorting was approximately 98%. A total of 1×10^5 CD34$^+$ cells were resuspended in 100 μL of DPBS and were intravenously injected into NSG mice (Jackson Laboratory). Three-week-old mice were sublethally irradiated with 300 cGy of total body irradiation for 24 h before intravenous injection of CB CD34$^+$ cells. At 8–12 weeks after the cell injection, FACS analysis was performed to confirm human cell engraftment using PB, BM, thymus, and spleen. Mice were monitored daily for symptoms of GVHD, including a ruffled coat, hunched back, weakness, and reduced motility. Once injected animals showed signs, they were sacrificed. In the absence of these signs of stress, mice were analyzed over 23 weeks after transplantation. All experiments were performed as described previously (Lee et al., 2014). Mice that did not show GVHD and showed a stable engraftment of human CD45 cells were used to monitor immune rejection by HLA types.

Flow Cytometry Analysis and IHC of Human Immune Cells in Reconstructed Humanized Mice

To analyze human hematopoietic cells, cells from the PB, BM, spleen, and thymus of reconstituted humanized mice were briefly resuspended in 100 μL of rinsing buffer and incubated with human antibodies. APC-conjugated mouse anti-human CD45 (BD), fluorescein isothiocyanate (FITC)-conjugated rat anti-mouse CD45 (BD), PerCP-conjugated mouse anti-human CD45RA (BioLegend), FITC-conjugated mouse anti-human CD45RO (BioLegend), PE-conjugated mouse anti-human CD19 (BioLegend), mouse anti-human TCRαβ (BioLegend), APC-conjugated mouse anti-human CD4 (BD), PE-conjugated mouse anti-human CD8 (BD), FITC-conjugated mouse anti-human CD33 (BD), rabbit anti-human MHC class I (Abcam), and mouse anti-human MHC class II (Abcam) were used as a primary antibodies. Flow cytometry data were analyzed using appropriate controls with proper isotype-matched immunoglobulin G and unstained controls. After washing, the resulting cells were analyzed on a BD Accuri C6 Plus Flow cytometer (BD) and data were evaluated with the CFlow software. IHC in tissues, including Beriplast (Avin Darou) was conducted as described previously (Jun et al., 2019). Sections were blocked for endogenous peroxidase activity and incubated with mouse anti-human STEM121 (Takara) and anti-human osteocalcin (Abcam) antibodies. Also, to stain CD4 and CD8 cells in the thymus of the humanized mouse, anti-CD4 (Abcam) and anti-CD8 antibodies (Abcam) were used. For confirmation of human engraftment, BM was subjected to hematoxylin and eosin staining.

Statistical Analysis

All results are presented as the mean ± SEM. Statistical analyses were performed with the Mann-Whitney U test and chi-square test for comparisons between two groups. Values of $p < 0.05$ were considered to denote statistical significance. The GraphPad Prism v.4 software (GraphPad software) was used for statistical analysis.

SUPPLEMENTAL INFORMATION

Supplemental Information can be found online at https://doi.org/10.1016/j.stemcr.2020.05.005.

AUTHOR CONTRIBUTIONS

D.R.L. conceived the project. J.E.L., J.Y.L., C-H.P., and D.R.L. designed the experiments. J.H.E. performed SCNT experiments. J.E.L. performed human PSC derivation experiments. J.E.L., C-H.P., S.K.J., and J.S.L. conducted stem cell experiments. J.Y.L., A-R.H., H.S.S., J.H.I., D-W.S., T.C., K.H., and D.R.H. conducted humanized mice experiments and immune analysis. J.H.E., J.E.L., and T.K.Y. performed human oocyte experiments. J.E.L., J.Y.L., and D.R.L. wrote the manuscript.

ACKNOWLEDGMENTS

We thank Drs. Jung-Il Lee and Seung-Young Go for technical help for humanized mouse project. This research is supported by the Bio & Medical Technology Development Program (2015M3A9C6028961, 2017M3A9F8072235, and 2017M3A 9C6061284) and Basic Science Research Program (2019 R1A2C2005681) of NRF and MSICT of Republic of Korea.

Received: April 24, 2020
Revised: May 5, 2020
Accepted: May 5, 2020
Published: June 4, 2020

REFERENCES

Chidgey, A.P., Layton, D., Trounson, A., and Boyd, R.L. (2008). Tolerance strategies for stem-cell-based therapies. Nature *453*, 330–337.

Cho, M.S., Lee, Y.E., Kim, J.Y., Chung, S., Cho, Y.H., Kim, D.S., Kang, S.M., Lee, H., Kim, M.H., Kim, J.H., et al. (2008). Highly efficient and large-scale generation of functional dopamine neurons from human embryonic stem cells. Proc. Natl. Acad. Sci. U S A *105*, 3392–3397.

Chung, Y.G., Eum, J.H., Lee, J.E., Shim, S.H., Sepilian, V., Hong, S.W., Lee, Y., Treff, N.R., Choi, Y.H., Kimbrel, E.A., et al. (2014). Human somatic cell nuclear transfer using adult cells. Cell Stem Cell *14*, 777–780.

Chung, Y.G., Matoba, S., Liu, Y., Eum, J.H., Lu, F., Jiang, W., Lee, J.E., Sepilian, V., Cha, K.Y., Lee, D.R., et al. (2015). Histone demethylase expression enhances human somatic cell nuclear transfer efficiency and promotes derivation of pluripotent stem cells. Cell Stem Cell *17*, 758–766.

Cohen, C.J., Zhao, Y., Zheng, Z., Rosenberg, S.A., and Morgan, R.A. (2006). Enhanced antitumor activity of murine-human hybrid T-cell receptor (TCR) in human lymphocytes is associated with improved pairing and TCR/CD3 stability. Cancer Res. *66*, 8878–8886.

Gurdon, J.B. (1962). The developmental capacity of nuclei taken from intestinal epithelium cells of feeding tadpoles. J. Embryol. Exp. Morphol. *10*, 622–640.

Jun, S.M., Park, M., Lee, J.Y., Jung, S., Lee, J.E., Shim, S.H., Song, H., and Lee, D.R. (2019). Single cell-derived clonally expanded mesenchymal progenitor cells from somatic cell nuclear transfer-derived pluripotent stem cells ameliorate the endometrial function in the uterus of a murine model with Asherman's syndrome. Cell Prolif. *52*, e12597.

Kato, Y., Tani, T., Sotomaru, Y., Kurokawa, K., Kato, J., Doguchi, H., Yasue, H., and Tsunoda, Y. (1998). Eight calves cloned from somatic cells of a single adult. Science *282*, 2095–2098.

Kooreman, N.G., de Almeida, P.E., Stack, J.P., Nelakanti, R.V., Diecke, S., Shao, N.Y., Swijnenburg, R.J., Sanchez-Freire, V., Matsa, E., Liu, C., et al. (2017). Alloimmune responses of humanized mice to human pluripotent stem cell therapeutics. Cell Rep. *20*, 1978–1990.

Lanza, R.P., Cibelli, J.B., and West, M.D. (1999). Prospects for the use of nuclear transfer in human transplantation. Nat. Biotechnol. *17*, 1171–1174.

Lee, A.R., Hong, K., Choi, S.H., Park, C., Park, J.K., Lee, J.I., Bang, J.I., Seol, D.W., Lee, J.E., and Lee, D.R. (2019). Anti-apoptotic regulation contributes to the successful nuclear reprogramming using cryopreserved oocytes. Stem Cell Reports *12*, 545–556.

Lee, J.E., Chung, Y.G., Eum, J.H., Lee, Y., and Lee, D.R. (2016). An efficient SCNT technology for the establishment of personalized and public human pluripotent stem cell banks. BMB Rep. *49*, 197–198.

Lee, J.Y., Park, S., Han, A.R., Lim, J., Min, W.S., and Kim, H.J. (2014). High ALDHdim-expressing CD34+CD38− cells in leukapheresed peripheral blood is a reliable guide for a successful leukemic xenograft model of acute myeloid leukemia. Oncol. Rep. *32*, 1638–1646.

Liu, Z., Cai, Y., Wang, Y., Nie, Y., Zhang, C., Xu, Y., Zhang, X., Lu, Y., Wang, Z., Poo, M., et al. (2018). Cloning of macaque monkeys by somatic cell nuclear transfer. Cell *172*, 881–887.e7.

Loh, Y.H., Agarwal, S., Park, I.H., Urbach, A., Huo, H., Heffner, G.C., Kim, K., Miller, J.D., Ng, K., and Daley, G.Q. (2009). Generation of induced pluripotent stem cells from human blood. Blood *113*, 5476–5479.

Mandai, M., Watanabe, A., Kurimoto, Y., Hirami, Y., Morinaga, C., Daimon, T., Fujihara, M., Akimaru, H., Sakai, N., Shibata, Y., et al. (2017). Autologous induced stem-cell-derived retinal cells for macular degeneration. N. Engl. J. Med. *376*, 1038–1046.

Matoba, S., Liu, Y., Lu, F., Iwabuchi, K.A., Shen, L., Inoue, A., and Zhang, Y. (2014). Embryonic development following somatic cell nuclear transfer impeded by persisting histone methylation. Cell *159*, 884–895.

Morishima, S., Shiina, T., Suzuki, S., Ogawa, S., Sato-Otsubo, A., Kashiwase, K., Azuma, F., Yabe, T., Satake, M., Kato, S., et al. (2018). Evolutionary basis of HLA-DPB1 alleles affects acute GVHD in unrelated donor stem cell transplantation. Blood *131*, 808–817.

Okita, K., Matsumura, Y., Sato, Y., Okada, A., Morizane, A., Okamoto, S., Hong, H., Nakagawa, M., Tanabe, K., Tezuka, K., et al. (2011). A more efficient method to generate integration-free human iPS cells. Nat. Methods *8*, 409–412.

Pappas, D.J., Gourraud, P.A., Le Gall, C., Laurent, J., Trounson, A., DeWitt, N., and Talib, S. (2015). Proceedings: human leukocyte antigen haplo-homozygous induced pluripotent stem cell haplobank modeled after the California population: evaluating matching in a multiethnic and admixed population. Stem Cells Transl. Med. *4*, 413–418.

Practice Committees of American Society for Reproductive Medicine; Society for Assisted Reproductive Techonology (2013). Mature oocyte cryopreservation: a guideline. Fertil. Steril. *99*, 37–43.

Rim, Y.A., Park, N., Nam, Y., Ham, D.S., Kim, J.W., Ha, H.Y., Jung, J.W., Jung, S.M., Baek, I.C., Kim, S.Y., et al. (2018). Recent progress of national banking project on homozygous HLA-typed induced pluripotent stem cells in South Korea. J. Tissue Eng. Regen. Med. *12*, e1531–e1536.

Rodriguez-Osorio, N., Urrego, R., Cibelli, J.B., Eilertsen, K., and Memili, E. (2012). Reprogramming mammalian somatic cells. Theriogenology *78*, 1869–1886.

Ryan, J.M., Barry, F.P., Murphy, J.M., and Mahon, B.P. (2005). Mesenchymal stem cells avoid allogeneic rejection. J. Inflamm. *2*, 8.

Schwartz, S.D., Hubschman, J.P., Heilwell, G., Franco-Cardenas, V., Pan, C.K., Ostrick, R.M., Mickunas, E., Gay, R., Klimanskaya, I., and Lanza, R. (2012). Embryonic stem cell trials for macular degeneration: a preliminary report. Lancet *379*, 713–720.

Shultz, L.D., Saito, Y., Najima, Y., Tanaka, S., Ochi, T., Tomizawa, M., Doi, T., Sone, A., Suzuki, N., Fujiwara, H., et al. (2010). Generation of functional human T-cell subsets with HLA-restricted immune responses in HLA class I expressing NOD/SCID/IL2r gamma(null) humanized mice. Proc. Natl. Acad. Sci. U S A *107*, 13022–13027.

Song, W.K., Park, K.M., Kim, H.J., Lee, J.H., Choi, J., Chong, S.Y., Shim, S.H., Del Priore, L.V., and Lanza, R. (2015). Treatment of macular degeneration using embryonic stem cell-derived retinal pigment epithelium: preliminary results in Asian patients. Stem Cell Reports *4*, 860–872.

Sugita, S., Iwasaki, Y., Makabe, K., Kamao, H., Mandai, M., Shiina, T., Ogasawara, K., Hirami, Y., Kurimoto, Y., and Takahashi, M. (2016a). Successful transplantation of retinal pigment epithelial cells from MHC homozygote iPSCs in MHC-matched models. Stem Cell Reports *7*, 635–648.

Sugita, S., Iwasaki, Y., Makabe, K., Kimura, T., Futagami, T., Suegami, S., and Takahashi, M. (2016b). Lack of T cell response to iPSC-derived retinal pigment epithelial cells from HLA homozygous donors. Stem Cell Reports *7*, 619–634.

Tachibana, M., Amato, P., Sparman, M., Gutierrez, N.M., Tippner-Hedges, R., Ma, H., Kang, E., Fulati, A., Lee, H.S., Sritanaudomchai, H., et al. (2013). Human embryonic stem cells derived by somatic cell nuclear transfer. Cell *153*, 1228–1238.

Taylor, C.J., Peacock, S., Chaudhry, A.N., Bradley, J.A., and Bolton, E.M. (2012). Generating an iPSC bank for HLA-matched tissue transplantation based on known donor and recipient HLA types. Cell Stem Cell *11*, 147–152.

Turner, M., Leslie, S., Martin, N.G., Peschanski, M., Rao, M., Taylor, C.J., Trounson, A., Turner, D., Yamanaka, S., and Wilmut, I. (2013). Toward the development of a global induced pluripotent stem cell library. Cell Stem Cell *13*, 382–384.

Wakayama, T., Perry, A.C., Zuccotti, M., Johnson, K.R., and Yanagimachi, R. (1998). Full-term development of mice from enucleated oocytes injected with cumulus cell nuclei. Nature *394*, 369–374.

Wilmut, I., Schnieke, A.E., McWhir, J., Kind, A.J., and Campbell, K.H. (1997). Viable offspring derived from fetal and adult mammalian cells. Nature *385*, 810–813.

Yamada, M., Johannesson, B., Sagi, I., Burnett, L.C., Kort, D.H., Prosser, R.W., Paull, D., Nestor, M.W., Freeby, M., Greenberg, E., et al. (2014). Human oocytes reprogram adult somatic nuclei of a type 1 diabetic to diploid pluripotent stem cells. Nature *510*, 533–536.

Yoon, T.K., Lee, D.R., Cha, S.K., Chung, H.M., Lee, W.S., and Cha, K.Y. (2007). Survival rate of human oocytes and pregnancy outcome after vitrification using slush nitrogen in assisted reproductive technologies. Fertil. Steril. *88*, 952–956.

Zhang, S.C., Wernig, M., Duncan, I.D., Brustle, O., and Thomson, J.A. (2001). In vitro differentiation of transplantable neural precursors from human embryonic stem cells. Nat. Biotechnol. *19*, 1129–1133.

Stem Cell Reports, Volume *15*

Supplemental Information

Cryopreserved Human Oocytes and Cord Blood Cells Can Produce Somatic Cell Nuclear Transfer-Derived Pluripotent Stem Cells with a Homozygous HLA Type

Jeoung Eun Lee, Ji Yoon Lee, Chang-Hwan Park, Jin Hee Eum, Soo Kyung Jung, A-Reum Han, Dong-Won Seol, Jin Saem Lee, Hyun Soo Shin, Jung Ho Im, Taehoon Chun, Kyungsoo Ha, Deok Rim Heo, Tae Ki Yoon, and Dong Ryul Lee

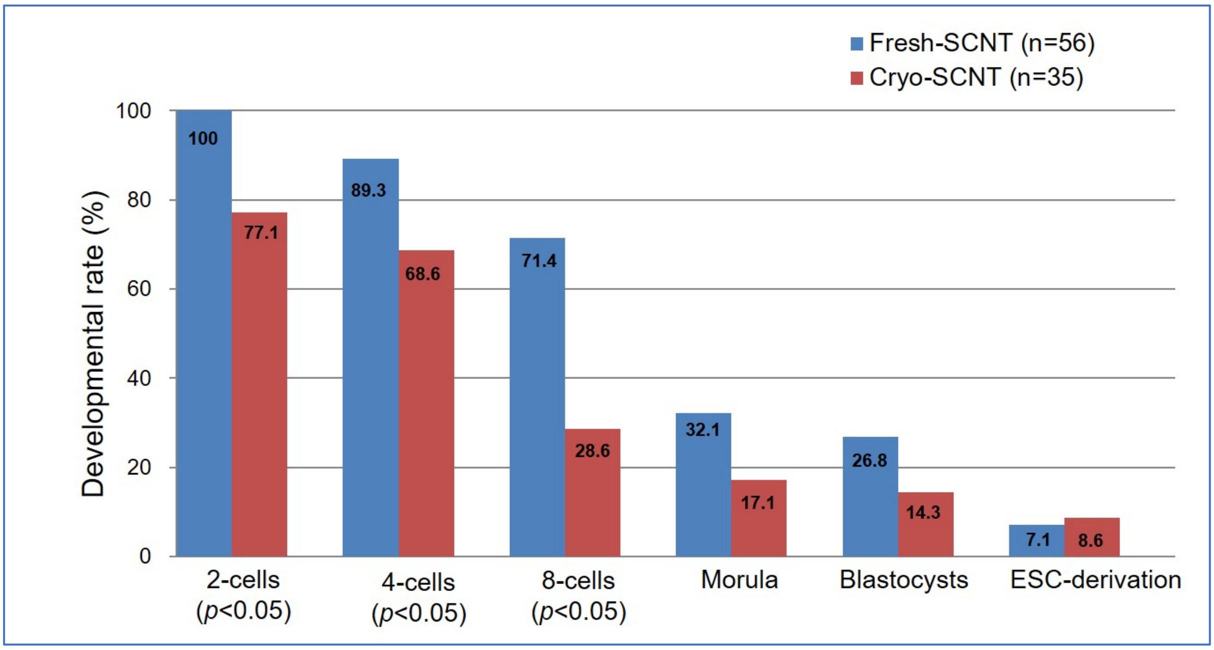

Figure S1. Comparison of embryonic development between fresh oocyte-based (Fresh)-somatic cell nuclear transfer (SCNT) and cryopreserved oocyte-based (Cryo)-SCNT (related to Figure 1)

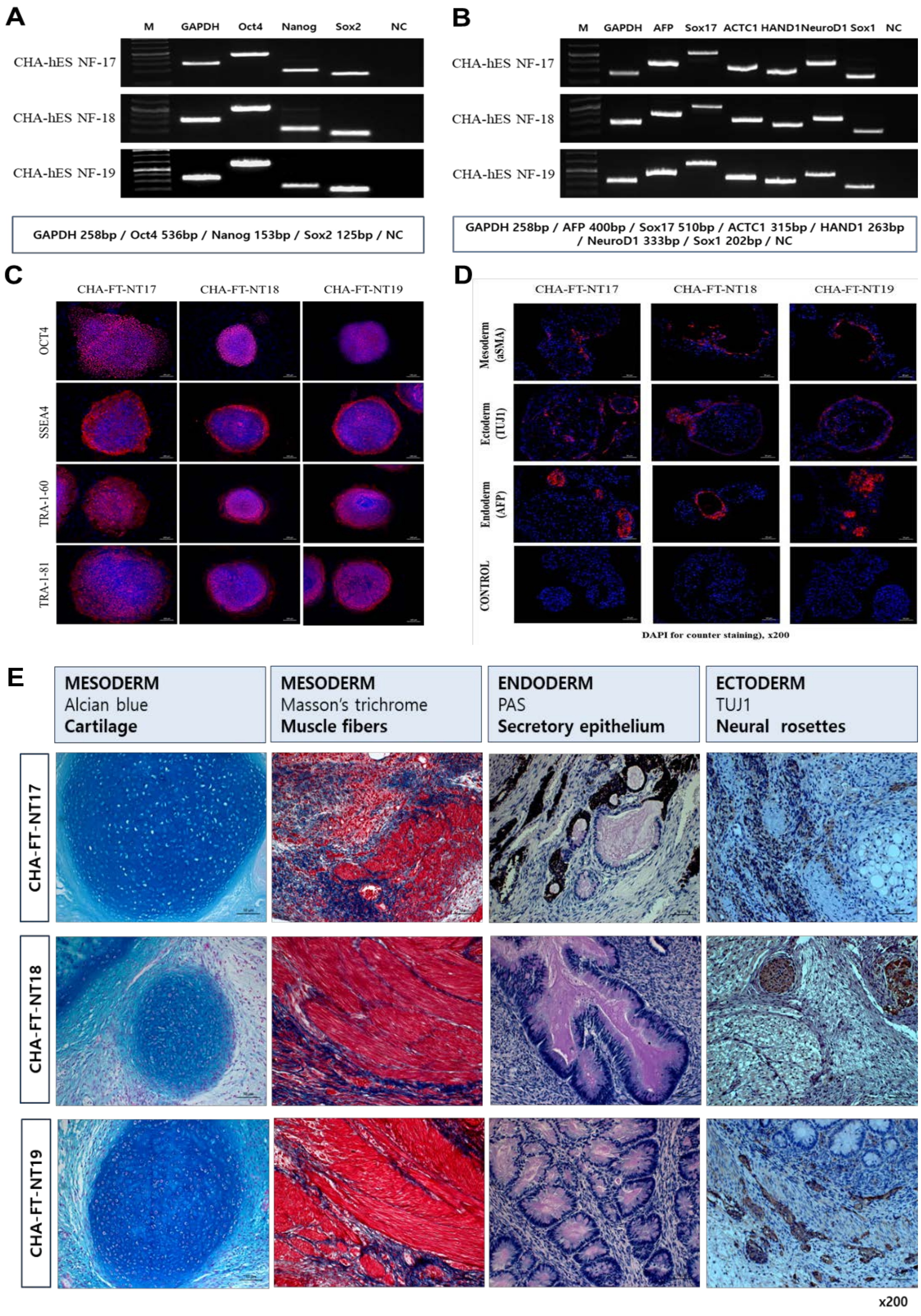

Figure S2. Characteristics of somatic cell nuclear transfer-derived pluripotent stem cells (SCNT-PSCs) obtained using cryopreserved human oocytes (related to Figure 2) To confirm the pluripotency of the

SCNT-PSC line derived from vitrified/warmed oocytes, their differentiation capacity was examined by *in vitro* EB formation. (A) RT-PCR showing the expression of pluripotency-related genes (*POU5F1*(*OCT4)*, *SOX2*, and *NANOG*) in SCNT-PSC colonies. (B) RT-PCR showing the expression of three germ layer-marker genes in embryonic bodies (EBs) of SCNT-PSCs. *GAPDH*: internal control; *SOX17* and *HAND1:* mesoderm ectoderm; *NEUROD1* and *SOX1*: ectoderm; *AFP* and *ACTC1*: endoderm; and N.C: negative control. (C) Expression of pluripotent stem cell markers (OCT4, SSEA4, TRA-1-60, and TRA-1-81) in SCNT-PSC colonies. The scale bars indicate 100μm. (D) Expression of markers of three germ layers in EBs of SCNT-PSCs, αSMA: mesoderm; TUJ1: ectoderm; AFP: endoderm; and control (no antibody). The scale bars indicate 50μm. (E) To confirm the pluripotency of the SCNT-PSC line derived from vitrified/warmed oocytes, their differentiation capacity was examined by *in vivo* teratoma assays. Teratoma confirmed the differentiation of somatic cell nuclear transfer-derived pluripotent stem cells (SCNT-PSCs) using cryopreserved human oocytes. From left, Alcian blue staining of mesoderm-derived cartilage, Masson’s trichrome staining of mesoderm-derived muscle fibers, periodic acid Schiff (PAS) staining of endoderm-derived secretory epithelium, and TUJ1 staining of ectoderm-derived neural rosettes. The scale bars indicate 50μm.

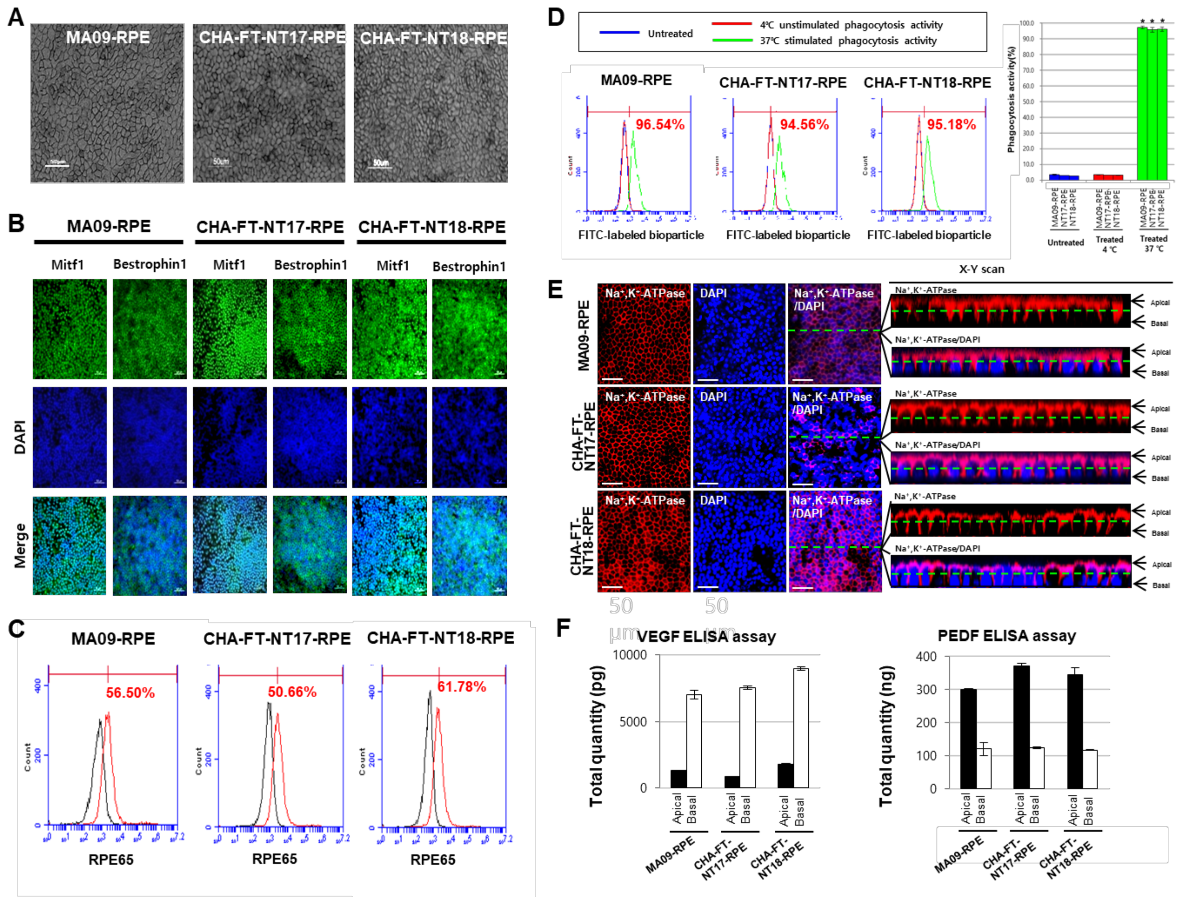

Figure S3. Characterization of somatic cell nuclear transfer-pluripotent stem cell-derived retinal pigmented epithelial cells (SCNT-PSC-RPEs) (related to Figure 3) (A) SCNT-PSC-RPEs (CHA-FT-NT17-RPEs and CHA-FT-NT18-RPEs) and a well-characterized hESC line (MA09-RPEs) have shown typical RPE characteristics, such as pigmentation and cuboidal epithelial morphology in tissue culture. The scale bars indicate 50µm. (B, C) Compared to MA09-RPEs, SCNT-PSC-RPEs were similarly stained for hRPE markers, including MITF1 and BESTROPHIN1, and they expressed RPE65. The scale bars indicate 50µm. (D-F) The phagocytotic activity and polarity (Na^+, K^+-ATPase activity and expression of growth factors) of RPEs differentiated from PSCs were not different. The scale bars indicate 50µm.

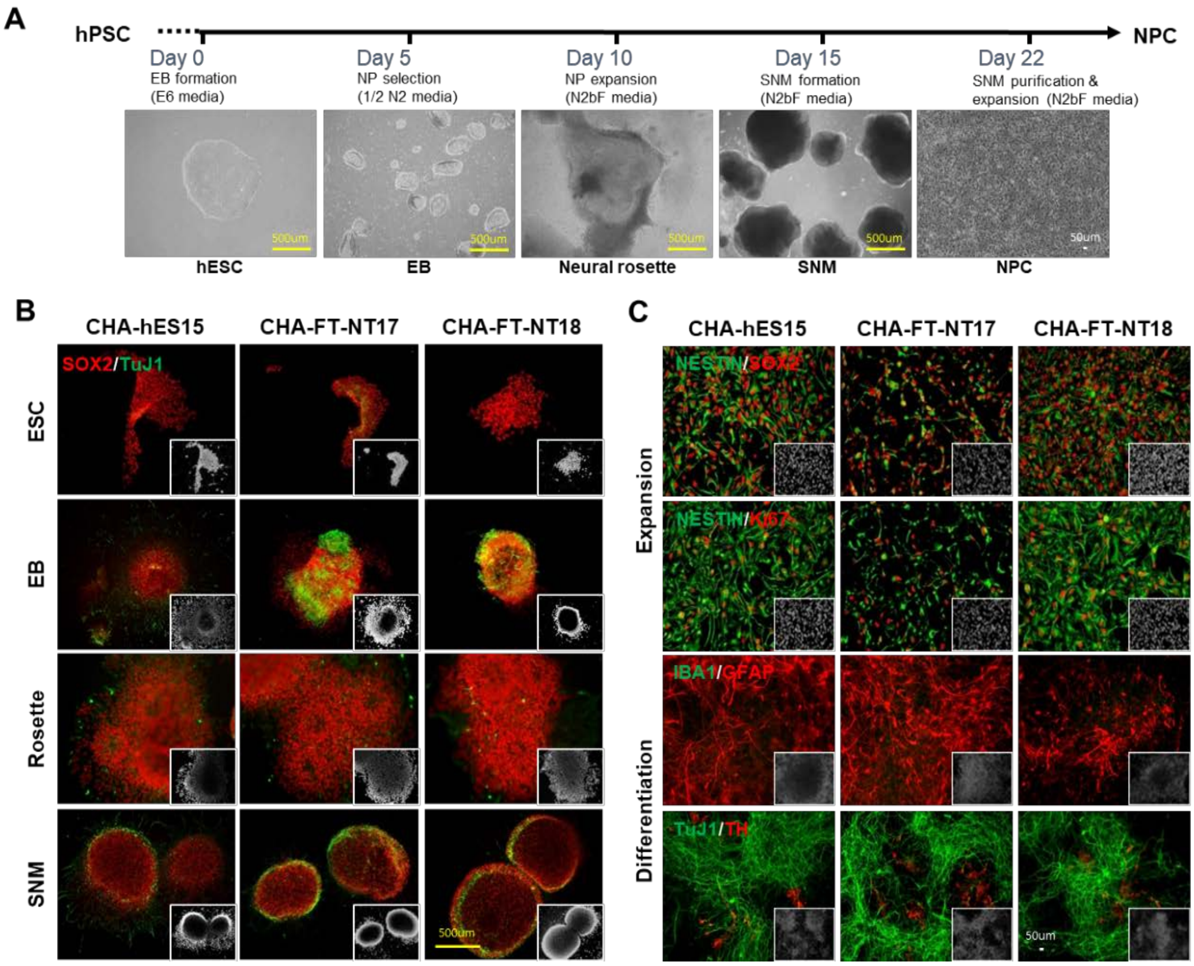

Figure S4. Characterization of somatic cell nuclear transfer-pluripotent stem cell-derived neural precursor cells (SCNT-PSC-NPCs) (related to Figure 3) (A) Schematic procedures for the *in vitro* differentiation of human PSCs into NPCs via spherical neural masses (SNMs). (B) Highly homogeneous SNMs were generated regardless of the human PSC line. Although some of the embryonic bodies expressed TuJ1, a neuronal marker, this expression disappeared in rosette-like structures and SNMs, indicating that rosettes and SNMs have pure NPC properties. (C) Differentiation characteristics of NPCs derived from various human PSCs. Most of the proliferating NPCs expressed NESTIN, SOX2 and Ki67. Upon removal of bFGF, NPCs differentiated into astrocytes and neurons as well as dopamine neurons. The yellow scale bars indicate 500µm. The white scale bars indicate 50µm.

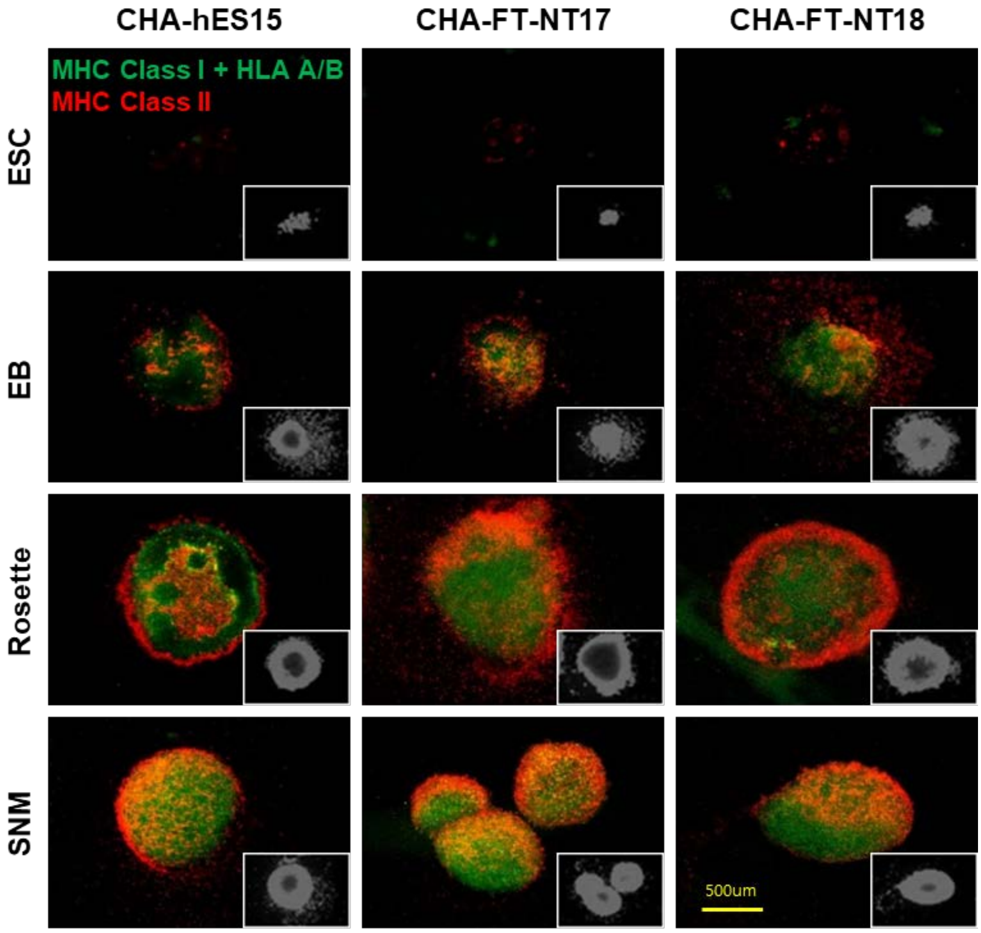

Figure S5. Expression of major histocompatibility antigens by differentiation stage in human pluripotent stem cells (PSCs) (related to Figure 4) MHC class I and II antigens were not expressed in PSCs regardless of the cell line, but their expression gradually increased with differentiation into the embryonic body (EB), neural rosette, and spherical neural mass (SNM) stages. The scale bar indicates 500μm.

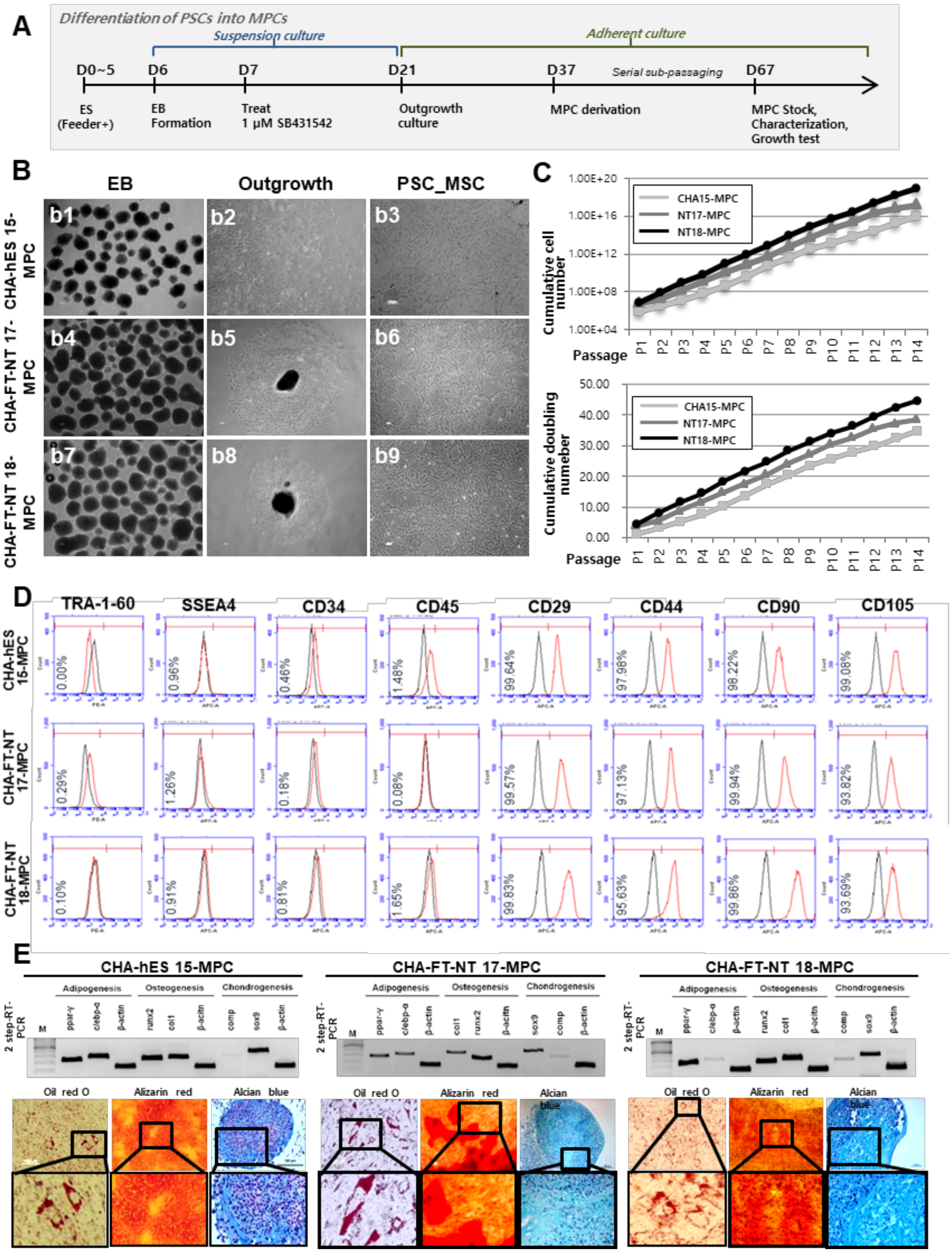

Figure S6. Generation and characterization of mesenchymal progenitor cells (MPCs) from human somatic cell nuclear transfer-pluripotent stem cells (SCNT-PSCs) (related to Figure 6) (A) Schema outlining the PSC-MPC differentiation protocol. The black horizontal line: time-line of days after initiation of differentiation. Days when a treatment was introduced are indicated (see Methods for details). From day 0 (D0) to day 6 (D6), human PSCs were cultured with MEF feeder cells for EB formation. SB431542 (1 μM) was added from D7 until D21 and then, these EBs were attached to the 6-well culture plate for outgrowth and expansion of MPCs until D37. Through serial subpassaging, matured and purified PSC-MPC were obtained and collected for the proliferation test, FACS analysis, and differentiation into specific-mesodermal lineages (adipocytes, osteocytes, and chondrocytes). (B) Representative morphology of human SCNT-PSC-derived MPCs (SCNT-PSC-MPCs) at different stages during the course of differentiation. (b1,b4,b7) Embryoid bodies (EBs) on day 1. (b2,b5,b8) Attachment and outgrowth of primitive MPCs on

day 14. (b3,b6,b9) The typical morphology of human SCNT-PSC-MPCs at passage 6. The scale bars indicate 100μm. (C) SCNT-PSC-MPC growth kinetics over long-term expansion. Graphs represent the cumulative cell number (upper) and cumulative population doubling level (CPDL) (bottom). Graphs are displayed from the passage in the initiation state of MPC-derivation to the present passage (P1 to P14). (d) Surface antigen profiling by FACS in SCNT-PSC-MPCs for TRA-1-60 and SSEA4 used as pluripotent markers, CD34 and CD45 used as hematopoietic markers, and CD29, CD44, CD90, and CD105 used as mesenchymal progenitor cell markers. (E) The transcription levels of the adipocyte genes, *PPAR-γ* and *C/EBP-α*, the osteocyte genes, *RUNX2* and *COL1*, and the chondrocyte genes, *sox9* and *comp* were measured by RT-PCR. β-actin was used as a loading control (upper). Differentiation ability of SCNT-PSC-MPCs to adipocytes, chondrocytes, and osteocytes (bottom). The lipid droplets in adipocytes were stained with Oil Red O. The calcium deposits in the intracellular region in osteocytes were stained with alizarin red solution. Mucopolysaccharides and acidic mucins expressed on the surface of the chondrocytes were stained with alcian blue solutions. The magnified images of each differentiation are presented in the bottom row. The scale bars indicate 100μm.

A

		HLA-A	HLA-B	HLA-C	DRB1	DQB1	DPB1
	SCNT-ES derived osteoblast	*33:03(A33)/*33:03(A33)	*44:03(B44)/*44:03(B44)	*14:03/*14:03	*13:.02/*13:02	*06:.04/*06:.04	*04:01/*04:01
Mismatched group for HLA-II DPB1	PBMC 2 (18-15)	*33:03(A33)/*33:03(A33)	*44:03(B44)/*58:01(B58)	*03:02/*14:.03	*13:02/*13:02	*06:04/*06:.09	*02:01/*02:01
	PBMC 3 (16-206)	*02:01(A2)/*33:03(A33)	*27:05(B27)/*44:03(B44)	*01:02/*14:.03	*01:01/*13:02	*05:01/*06:.04	*05:01/*05:01
	Matched group for HLA-I (A,B,C) and II (DRB1, DQB1)						Mismatched for DPB1
Matched group for HLA-II DPB1	PBMC 5 (16-203)	*03:01(A3)/*33:03(A33)	*27:05(B27)/*44:03(B44)	*02:02/*14:.03	*13:02/*15:.02	*06:01/*06:.04	*04:01/*14:01
	PBMC 6 (16-242)	*02:01(A2)/*33:03(A33)	*35:01(B7)/*44:03(B44)	*03:03/*14:.03	*08:03/*13:02	*06:01/*06:.04	*04:01/*19:01
	Matched group for HLA-I (A,B,C) and II (DRB1, DQB1)						Matched for DPB1, thus all matched

B

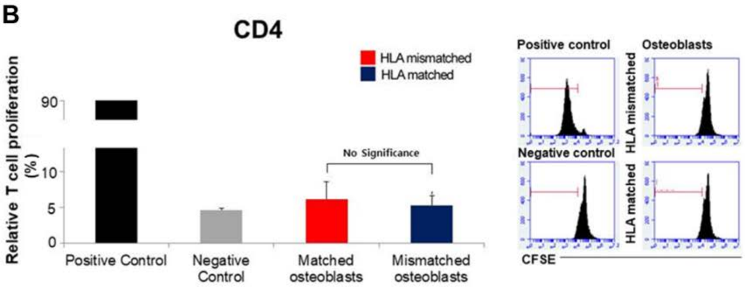

Figure S7. No significant difference was detected in CD4 T cell proliferation, when mismatched HLA-II, DPB1 was cocultured with homozygous SCNT-PSC-derived osteoblasts (related to Figure 6). (A) HLA genotyping data of PBMCs used in the present study. (B) CD4 T cell proliferation data showed that no significant difference by mismatched HLA-II DPB1 was shown in homozygous SCNT-PSC derived osteoblasts, suggesting a negligible risk of immune rejection of homozygous SCNT PSCs derived lineage cells. Each value is the average of 2 independent experiments (n=3 per experiment).

SUPPLMENTARY EXPERIMENTAL PROCEDURE

Characterization of human SCNT-PSCs

For *in vitro* differentiation assays, SCNT-PSCs were cultured in low-attachment dishes with ES C medium (without bFGF) for 2 weeks in order to obtain embryoid bodies (EBs), and then EBs were analyzed histologically. After fixing, washing, blocking, and permeabilization, EBs were incubated with primary antibodies at 4℃ overnight. After three washes with PBS containing 1% BSA, EBs were staine d with secondary antibodies and DAPI for 1 hour and observed under a fluorescence microscope. For t eratoma assays, approximately $1x10^5$ of undifferentiated SCNT-PSCs were injected into the testicle of a severe combined immunodeficiency (SCID) mouse (Chung et al., 2014). Also, karyotype analysis, genomic DNA fingerprinting, and mitochondrial DNA genotyping of three SCNT-PSC lines were performed usin g a standard protocol as previously described (Chung et al., 2014).

Differentiation into RPEs

RPE production from SCNT-PSCs (CHA-FT-NT17 and CHA-FT-NT18 as SCNT-PSCs) and ESCs (MA09) was performed using a well-established protocol as previously described (Lu et al., 2009). The pigmented RPE cells were passaged and harvested at passage 3 for characterization. To characterize, SCNT-PSC-derived RPEs were incubated with primary antibodies [anti-PAX6 (Millipore), anti-Bestrophin (Novus), anti-RPE65 (Abcam), anti-Mitf (Millipore), and anti-Sodium-Potassium exchange pump (Abcam) antibodies] diluted 1:100 in blocking solution overnight at 4℃. The cells were then incubated with Alexa Fluor 647-conjugated anti-rabbit IgG (GIBCO) and Alexa Fluor 488-conjugated anti-mouse IgG (GIBCO) for 1 hour at RT. For nuclear staining, cells were incubated with DAPI (Thermo) for 5 minutes; negative controls were incubated with secondary antibodies alone. Stained cells were observed under a Axiovert 200M inverted fluorescence microscope (Carl Zeiss). Flow cytometry analysis was used to measure the expression of RPE-specific markers. To test phagocytosis activity, SCNT-PSC-derived RPEs were treated with a fluorescent-labeled bio-particle™ (Vybrant® Phagocytosis assay kit, Life technologies) according to the manufacturer's instructions. Phagosomes were analyzed by flow cytometry using a FACSCalibur™ (BD). A minimum of 10,000 cells were analyzed for each sample. To confirm the polarized secretion of vascular endothelial growth factor (VEGF) from the basal side of RPE cells and pigment epithelium-derived factor (PEDF) from the apical side of RPE cells, SCNT-PSC-derived RPEs were seeded onto a 6 trans-well culture dish (Corning) and then differentiated to mature RPEs. The media from both the upper and lower reservoirs were collected after 30 days of culture. The amount of secreted proteins was measured by the Quantikine human VEGF ELISA kit (R&D systems) and the human PEDF ELISA kit (Biocompare) according to manufacturer's instruction. All assays were performed in triplicate.

Differentiation into neuronal lineage and characterization

The undifferentiated PSC lines (CHA-hES15 as conventional ESC; CHA-FT-NT17 and CHA-FT-NT18 as SCNT-PSCs) were maintained and differentiated into neuronal lineage as described previously (Cho et al., 2008). Briefly, EBs from human PSC colonies were differentiated to neural precursor cells (NPCs) via neural rosette-like structures and spherical neural masses (SNMs). Most of the SNMs derived from SCNT-PSCs and ESCs expressed NESTIN and SOX2, markers for neural precursor cells (NPCs). Most of the single cell-dissociated NPCs were NESTIN and SOX2 positive, and after 10 days, half of the differentiated cells were TUJ1 positive and the other half GFAP positive (Figure S5). Immunocytochemisty was performed as described previously (Kim et al., 2017). Cells were incubated with primary antibodies at 4°C overnight. The following primary antibodies were used: rabbit polyclonal antibodies, SOX2 (Sigma), NESTIN (BioLegend), tyrosine hydroxylase (TH) (Pel-Freez), neuron-specific class III beta-tubulin (TuJ1) (Covance); mouse monoclonal antibodies, TuJ1 (Covance), GFAP (ICN Biochem), Ki67 (Novocastra), IBA1 (FUJIFILM), MHC Class II (Abcam). MHC Class I (HLA A+HLA B, Abcam).

Differentiation into MPCs and their characterization

PSC-derived MPCs (from CHA-hES15, CHA-FT-NT17, and CHA-FT-NT18) were produced by the method as described previously (Jun et al., 2019). For flow cytometry analysis of cell surface markers, SCNT-PSCs and ESCs-derived MPCs were washed once with DPBS without Ca^{2+}/Mg^{2+} and fixed for 1 hour in a pre-chilled 4% PFA solution (EL Bio) at 4°C. A phycoerythrin (PE)-conjugated mouse anti-human TRA-1-60 (BD) and an allophycocyanine (APC)-conjugated mouse anti-human/mouse SSEA4 (R&D systems) were used as stemness markers; APC-conjugated mouse anti human CD34 (BD) and APC-conjugated mouse anti-human CD45 (BD) were used as hematopoietic markers; APC-conjugated mouse anti-human CD29 (BD), APC-conjugated mouse anti-human CD44 (BD), APC-conjugated mouse anti-human CD90 (BD), and APC-conjugated mouse anti-human CD105 (BD) were used as the MPC markers. After incubation and washing, cells were analyzed using an BD Accuri™ C6 Plus Flow cytometer equipped with the Cell Quest software (BD).

Differentiation into Adipocytes, Osteocytes, and Chondrocytse

For adipogenic differentiation, SCNT-PSCs and ESCs-derived MPCs were seeded onto 0.1% gelatin-coated (Sigma) 12-well tissue culture dish (Nunc) at a concentration of $2x10^5$ cells/well in MPC expansion media. When the cells were fully confluent, the adipogenic differentiation medium (StemPro® Adipogenesis Differentiation Kit, GIBCO) was added to induce adipocyte differentiation (21 days). Cells were stained with an Oil Red O solution (IHC world) according to the manufacturer's instructions. For osteogenic differentiation, osteogenic differentiation media (StemPro® Osteogenesis Differentiation Kit, GIBCO) was added to induce osteocyte differentiation (21 days). Cells were stained with an Alizarin red solution (IHC world) according to the manufacturer's instructions. For chondrogenic differentiation, MPCs were seed in a 15 mL conical tube (BD) at a concentration of $5x10^5$ cells, in chondrogenesis medium (StemPro® Chondrogenesis Differentiation Kit, GIBCO) for approximately 28 days. Differentiated cells were fixed and then embedded in paraffin. The sectioned samples were stained with an Alcian blue staining solution (IHC World) according to the manufacturer's instructions.

RT-PCR or Real-Time PCR of MPC-derived differentiated cells

PCR and real-time PCR were performed on a Bio-Rad thermal cycler (Bio-Rad) or on a Real-time PCR detection system (Bio-Rad). Primers of lineage-specific markers were designed using the Primer Express version 1.0 (PE Applied Biosystems) using RNA sequences obtained from GenBank. Primer sequences are as followed: adipocyte-related genes: *PPARG* (*PPAR-γ*; forward 5'-TGTCTCATAATGCCATCAGGTTTG-3', reverse 5'-GATAACGATGGTGATTTGTCTGTT-3', 224bp) and *CEBPA* (*C/EBP-α*; forward 5'-GCAAACTCACCGCTCCAATG-3', reverse 5'-TTAGGTTCCAAGCCCCAAGTC-3', 247bp), osteocyte-related genes: *RUNX2* (forward 5'-CCCCACGACAACCGCACCAT-3', reverse 5'CACTCCGGCCCACAAATCTC-3', 289bp) and *COL1A1* (*COL1*; forward 5'-AGAACATCACCTACCACTGC-3', reverse 5'-ATGTCCAAAGGTGCAATATC-3', 250bp), and chondrocyte-related genes: *SOX9* (forward 5'-TTCATGAAGATGACCGACGA-3', reverse 5'-CACACCATGAAGGCGTTCAT-3', 326bp), *COMP* (forward 5'-AACGCTGAAGTCACGCTCAC-3', reverse 5'-GGTAGCCAAAGATGAAGCCC-3', 244bp), *Alkaline phosphatase* (forward 5'- ACC ATT CCC ACG TCT TCA CAT TTG -3', reverse 5'- AGA CAT TCT CTC GTT CAC CGC C -3', 250bp), and *OSTEOCALCIN* (forward 5'- ATG AGA GCC CTC ACA CTC CTC -3', reverse 5'- GCC GTA GAA GCG CCG ATA GGC -3', 245bp). *ACTB* (*β-ACTIN*; forward 5'-TGAAGTGTGACGTGGACATC-3', reverse 5'-GGAGGAGCAATGATCTTGAT-3', 152bp)) and *GAPDH* (forward 5'- AGA AGG CTG GGG CTC ATT -3', reverse 5'- AGG GGC CAT CCA CAG TCT -3', 247bp) were used as a loading control.

SUPPLMENTARY REFERENCES

Cho, M.S., Lee, Y.E., Kim, J.Y., Chung, S., Cho, Y.H., Kim, D.S., Kang, S.M., Lee, H., Kim, M.H., Kim, J.H., *et al.* (2008). Highly efficient and large-scale generation of functional dopamine neurons from human embryonic stem cells. Proc Natl Acad Sci U S A *105*, 3392-3397.

Chung, Y.G., Eum, J.H., Lee, J.E., Shim, S.H., Sepilian, V., Hong, S.W., Lee, Y., Treff, N.R., Choi, Y.H., Kimbrel, E.A., *et al.* (2014). Human somatic cell nuclear transfer using adult cells. Cell Stem Cell *14*, 777-780.
Jun, S.M., Park, M., Lee, J.Y., Jung, S., Lee, J.E., Shim, S.H., Song, H., and Lee, D.R. (2019). Single cell-derived clonally expanded mesenchymal progenitor cells from somatic cell nuclear transfer-derived pluripotent stem cells ameliorate the endometrial function in the uterus of a murine model with Asherman's syndrome. Cell Prolif *52*, e12597.
Kim, S.M., Lim, M.S., Lee, E.H., Jung, S.J., Chung, H.Y., Kim, C.H., and Park, C.H. (2017). Efficient Generation of Dopamine Neurons by Synthetic Transcription Factor mRNAs. Mol Ther *25*, 2028-2037.
Lu, B., Malcuit, C., Wang, S., Girman, S., Francis, P., Lemieux, L., Lanza, R., and Lund, R. (2009). Long-term safety and function of RPE from human embryonic stem cells in preclinical models of macular degeneration. Stem Cells *27*, 2126-2135.
